# Influence of conventional hydrogen bonds in the intercalation of phenanthroline derivatives with DNA: The important role of the sugar and phosphate backbone

**DOI:** 10.1002/jcc.26836

**Published:** 2022-03-17

**Authors:** Ángel Sánchez‐González, Pierre Grenut, Adrià Gil

**Affiliations:** ^1^ BioISI—Biosystems and Integrative Sciences Institute, Departamento de Química e Bioquímica, Faculdade de Ciências Universidade de Lisboa, Campo Grande Lisbon Portugal; ^2^ ARAID Foundation Zaragoza Spain; ^3^ Departamento de Química Inorgánica Instituto de Síntesis Química y Catálisis Homogénea (ISQCH) CSIC‐Universidad de Zaragoza, c/ Pedro Cerbuna 12 Zaragoza Spain

**Keywords:** DFT‐D, DNA, phenanthroline derivatives, PM6‐DH2, weak interactions

## Abstract

The influence of hydrogen bonds in model intercalated systems between guanine‐cytosine and adenine‐thymine DNA base pairs (bps) was analyzed with the popular intercalator 1,10‐phenanthroline (phen) and derivatives obtained by substitution with —OH and —NH_2_ groups in positions 4 and 7. Semiempirical and Density Functional Theory (DFT) methods were used both including dispersion effects: PM6‐DH2, M06‐2X and B3LYP‐D3 along with the recently developed near linear‐scaling coupled cluster method DLPNO‐CCSD(T) for benchmark calculations. Our results given by QTAIM and non‐covalent interaction analysis confirmed the existence of hydrogen bonds created by —OH and —NH_2_. The trends in the energy decomposition analysis for the interaction energy, Δ*E*_int_, showed that the Δ*E*_elstat_ contributions are equal or even a little bit higher than the values for Δ*E*_disp_. Such important Δ*E*_elstat_ attractive contribution comes mainly from the conventional hydrogen bonds formed by —OH and —NH_2_ functional groups with DNA not only with bps but specially with the sugar and phosphate backbone. This behavior is very different from that of phen and other classical intercalators that cannot form conventional hydrogen bonds, where the Δ*E*_disp_ is the most important attractive contribution to the Δ*E*_int_. The inclusion of explicit water molecules in molecular dynamics simulations showed, as a general trend, that the hydrogen bonds with the bps disappear during the simulations but those with the sugar and phosphate backbone remain in time, which highlights the important role of the sugar and phosphate backbone in the stabilization of these systems.

## Graphical abstract

Hydrogen bond formation between phenanthroline derivatives and DNA sugar and phosphate backbone modulates the nature of the interaction from dispersion to electrostatic. Despite QM methods works usually with reduced models, the consideration of the sugar and phosphate backbone results mandatory to analyze the behavior of these systems where the intercalator may form conventional hydrogen bonds.

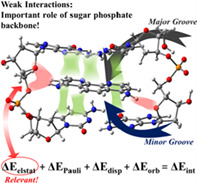

## 1 INTRODUCTION

The antitumoral effects of some metal complexes have arose a great interest for treatment of cancer disease.^1^, ^2^, ^3^ The cytotoxic properties of such complexes have allowed to develop several effective treatments against certain types of cancer as breast, testicular, or ovarian cancers.^1^, ^2^, ^3^ However, some of these treatments remain aggressive and are systematically at the origin of side effects. Therefore, more research in this field to reduce such aggressive side effects is a scientific multidisciplinary challenge. Indeed, the origin of the cytotoxicity of these complexes is globally known, they interact with the nuclear duplex DNA and produce the inhibition of its replication/transcription and thus, the inhibition of the multiplication of the cancer cells.^4^, ^5^ However the knowledge on how the molecule interacts with DNA, the induced structural changes that rise up when this interaction is produced, or the explanation of the perturbation of biological processes by these complexes are still dark aspects that need to be enlighten. Thus, one of the challenges of the scientific community related to this field is to discover how the ligands of metal complexes interact with DNA and how such interaction yields cytotoxic effects to better understand and improve the efficacy of chemotherapy treatments against cancer.

**FIGURE 1:**
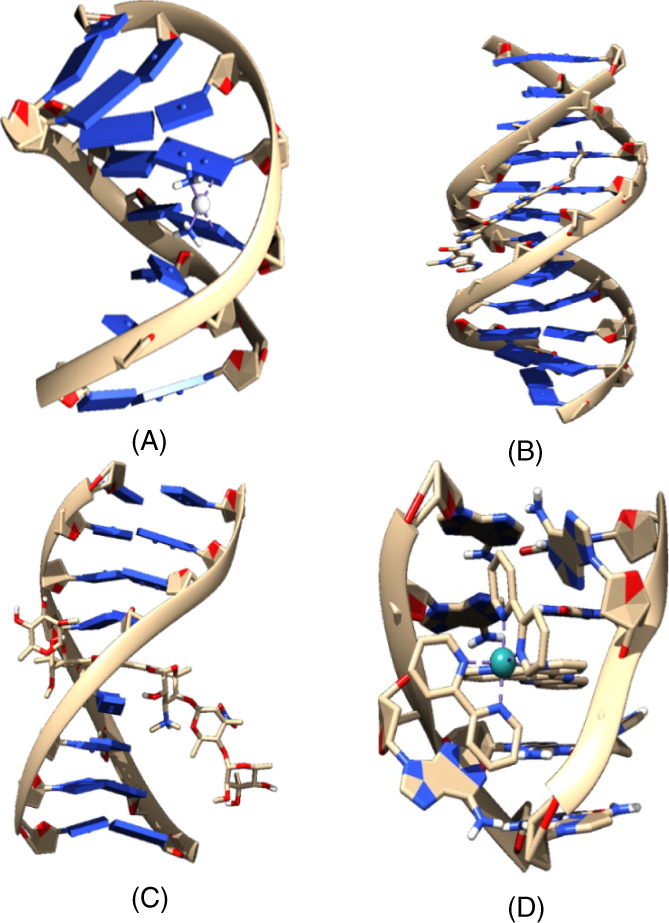
Structural representation for the interaction between any small molecule and duplex DNA. (A) Cross‐linking (PDB ID:1au5), (B) mg binding (PDB ID: 448d), (C) intercalation (PDB ID: 1n37), and (D) insertion (PDB ID: 4e1u)

Simulation and experimental techniques^6^, ^7^ have allowed to categorize two families of molecules that interact with DNA (see Figure 1). That is, the groove binders and the intercalators of DNA. The groove binders interact with the major groove, from now MG, and minor groove, from now mg, of the DNA by forming covalent bonds through cross‐links or by means of weak interactions. On the other hand, the intercalators of the DNA are included between adjacent base pairs (bps). Finally, there is a last mode of interaction called insertion that occurs when some small molecule is included between 2 bps of DNA that are not matched.^6^, ^8^ In such case, any flat molecule interacts from the mg side and ejects the mismatched DNA bases and such flat molecule acts as its π‐stacking replacement (see Figure 1). The interactions of the groove binders and intercalators result in changes in parameters of the structure of the duplex DNA, which are the origin of the therapeutic effects.^9^, ^10^

**SCHEME 1:**
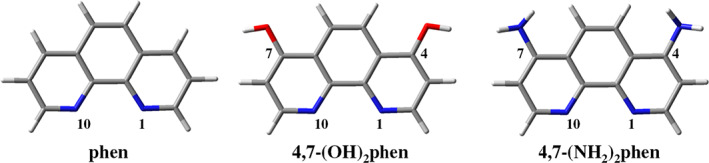
Phen and phen derivatives studied in this work

Many studies focus on the incorporation of the 1,10‐phenanthroline (phen) ligand and derivatives (see Scheme 1) in metal complexes having such systems significant antitumoral activity.^11^, ^12^, ^13^, ^14^, ^15^ Among the different interactions of the metal complexes with DNA, intercalation is an important binding mode^16^ in the use of these metal complexes for chemotherapy treatments and this mode of interaction is affected by the planarity of the ligand, type of donor atom and metal coordination geometry.^17^ Recent experimental works on the interaction of metal complexes including phen ligands with DNA have been consistent with the intercalation of this flat ligand between DNA bps.^12^, ^18^ Linear dichroism, viscosity, and NOESY and NMR spectra also showed that methylated derivatives of phen and dipyridol[3,2‐a:2′,3′‐c]phenazine (dppz) intercalated between DNA bps.^14^, ^19^ Finally, latter determination of crystal structures have given very important information about the intercalation of dppz ligand of [Ru(tap)_2_(dppz)]^2+^, [Ru(bpy)_2_(dppz)]^2+^, and [Ru(phen)_2_(dppz)]^2+^ between bps of duplex DNA.^8^, ^20^, ^21^, ^22^

On the other hand, it has been found in the bibliography^6^, ^23^, ^24^, ^25^, ^26^ that the process of intercalation needs prior formation of some non‐covalent mg binding system. Such process is governed by kinetics. That is, while mg is a very fast process (tenths of milliseconds), the intercalation process is slower (few milliseconds). Thus, the interaction between any small molecule and DNA is best described as some reversible kinetic equilibrium defining the residence time of such interaction.^27^ In general, the favored direction of the equilibrium is related to the strength of the formed hydrogen bonds and/or other weak interactions.^28^ The entropic and steric factors and desolvation energies have been also found to play an important role in the kinetics of the process.^6^, ^23^, ^24^, ^25^, ^26^ In this sense, the driving force for the intercalation may be related to the energy derived from the removal of the small molecule from the aqueous medium together with the intrinsic contributions to the interaction between the small molecule and DNA (Pauli, van der Waals, electrostatic and polarization).^29^, ^30^, ^31^, ^32^, ^33^, ^34^, ^35^, ^36^ Thus, since the cytotoxic effect of the intercalators depends on the time of residence of the drug between bps,^27^ whereas mg binding has not usually cytotoxic effects,^28^, ^37^ the design of efficient drugs should aim at favoring weak interactions to stabilize the intercalated state and destabilize the mg binding. Smart changes in the intercalators by means of the addition of different functional groups in number and position should increase the residence time of the drug between bps while preventing mg binding. Therefore, because the antitumoral activity of phen complexes has been already analyzed,^11^ and different studies have appeared in the bibliography dealing with the antitumoral activity of phen derivatives^38^, ^39^, ^40^ it is worth to study the effect of substitution of phen by means of groups capable to form conventional hydrogen bonds with DNA.

Several computational studies have been carried out over the last 20 years aiming at the modeling of the interaction of small molecules with DNA in which different approximations have been used.^23^, ^25^, ^26^, ^41^, ^42^, ^43^, ^44^, ^45^, ^46^, ^47^, ^48^, ^49^, ^50^, ^51^, ^52^, ^53^, ^54^, ^55^, ^56^, ^57^, ^58^ Nevertheless, it is possible to put the computational treatment of the interaction of drugs and DNA into three main sets. The first one would include large systems in which the interaction is studied by means of force fields derived from molecular mechanics (MM) or by means of classical molecular dynamics (MD). The models for such systems are usually long chains of DNA that go from decamers to hexadecamers along with the drug with/without explicit solvent and some examples are the works of Trieb et al.,^41^ Mukherjee et al.,^23^ Robertazzi et al.,^42^ Vargiu et al.,^43^ Galindo‐Murillo et al.,^44^ Sasikala et al.,^25^ or Franco et al.^26^ On the other hand, we have the accurate treatment of the electronic structure by means of quantum mechanics (QM) methods. However, such high‐level treatment (MP2 or DFT including dispersion corrections) implies the reduction of the model and such studies have used the three‐body model consisting of two bases (one base pair) and the drug (flat ligand). Some examples of this treatment are the seminal works of Bondarev et al.,^45^ or Rĕha et al.^46^ and more recently the works of Kumar et al.,^47^ El‐Gogary et al.,^48^, ^49^ Li et al.,^50^ or Hazarika et al.,^51^ the latter still used this simplest three‐body approach in 2011. Finally, between these two treatments we have other situations in the middle, which are in the third set. Indeed, we have some approaches in the bibliography in which models of two bps without (sandwich model),^56^ or with (ring model),^56^ the sugars and phosphates and the studied drugs are taken into account with several computational methods (MP2, DFT including dispersion corrections, TD‐DFT, or QM/MM). The examples for this last set are the studies of Xiao et al.,^52^ Langner et al.,^53^ Hill et al.,^54^ Ambrosek et al.,^55^ Biancardi et al.,^56^ Deepa et al.,^58^ or even our previous works on this topic.^30^, ^31^, ^32^, ^33^, ^35^, ^36^ Thus, taking into account the interest of using intercalators for a range of medical applications, a structural comprehension of the interactions of such drugs with DNA results important.^59^ On the other hand, it is also desirable to gain insight on how to tune the activity of such drugs by means of the introduction of substituents, which are able to form conventional hydrogen bonds with DNA when they are intercalated between bps.

Thus, the aim of this work is to carry out computational calculations to analyze the effects that the inclusion of —OH and —NH_2_ groups in phen, which are able to from conventional hydrogen bonds and strength the interaction, produce in the intercalation process by considering the so‐called ring models^56^ with not only two bps but also the sugar and phosphate backbone. This model is one of the most advanced models for QM calculations on this intercalating systems since the seminal studies by Bondarev et al.,^45^ and Rĕha et al.^46^ took into account the simplest three‐body models consisting of the two bases of the DNA base pair and the intercalator, whereas the so‐called sandwich model^56^ considers the two bps and the intercalator but does not take into account the sugar and phosphate backbone which is crucial to explain the interaction for intercalating systems that are able to form conventional hydrogen bonds as we will see in our present study. Thus, we will show in our work that consideration of the seminal three‐body models and even sandwich models to perform QM studies to analyze the electronic structures and the behavior of intercalators that may produce conventional hydrogen bonds is not enough and would lead to erroneous conclusions. Geometric parameters and energetics were analyzed and interaction properties were described in terms of the topology of electronic density, non‐covalent interaction (NCI) analysis and charge transfer, whereas the stabilization of the conventional hydrogen bonds in time were monitored by means of MD simulations. Previous computational studies have focused on the study of the interaction energy and the study of frontier orbitals of different DNA models by using intercalators in which the most important interaction to focus on was the π‐π stacking between bps and the intercalator.^45^, ^46^, ^47^, ^48^, ^49^, ^50^, ^51^, ^52^, ^53^, ^54^, ^55^, ^56^, ^57^, ^58^ However, the present study aims at trying to understand how derivatization of phen, a typical studied flat intercalator, by means of functional groups that are able to form conventional hydrogen bonds with DNA (—NH_2_ and —OH), affects in the intercalation process and gain insight on how the incorporation of such groups will modify the cytotoxicity. We shall use 4,7‐(OH)_2_phen and 4,7‐(NH_2_)_2_phen as intercalators for our study (see Scheme 1) and we will see how important is to take into account the sugar and phosphate backbone in the study because of the produced conventional hydrogen bonds with the intercalator. It means that even if our study is based on QM methods, which generally requires more reduced models than MM approaches, to analyze properties that are not possible to analyze with MM methods like molecular orbitals, QTAIM topologies and electron densities, Pauli repulsions in the interaction energy, and so forth, at least, we have to consider the ring model for a correct non‐dynamic QM study of the intercalated system, being the three‐body models (still used in 2011) and sandwich models, still used currently, not appropriate because they could lead to misleading conclusions. We expect that this work will help to understand how substitution of phen with —OH and —NH_2_ can tune the stability of the intercalation and thus affects the cytotoxicity of the ligand and how important is, even for QM calculations in which it is more difficult to work with large systems, to take into consideration the sugar and phosphate backbone of the DNA models used for the study of the intercalation of small molecules that may form conventional hydrogen bonds with DNA.

## 2 RESULTS AND DISCUSSION

**FIGURE 2:**
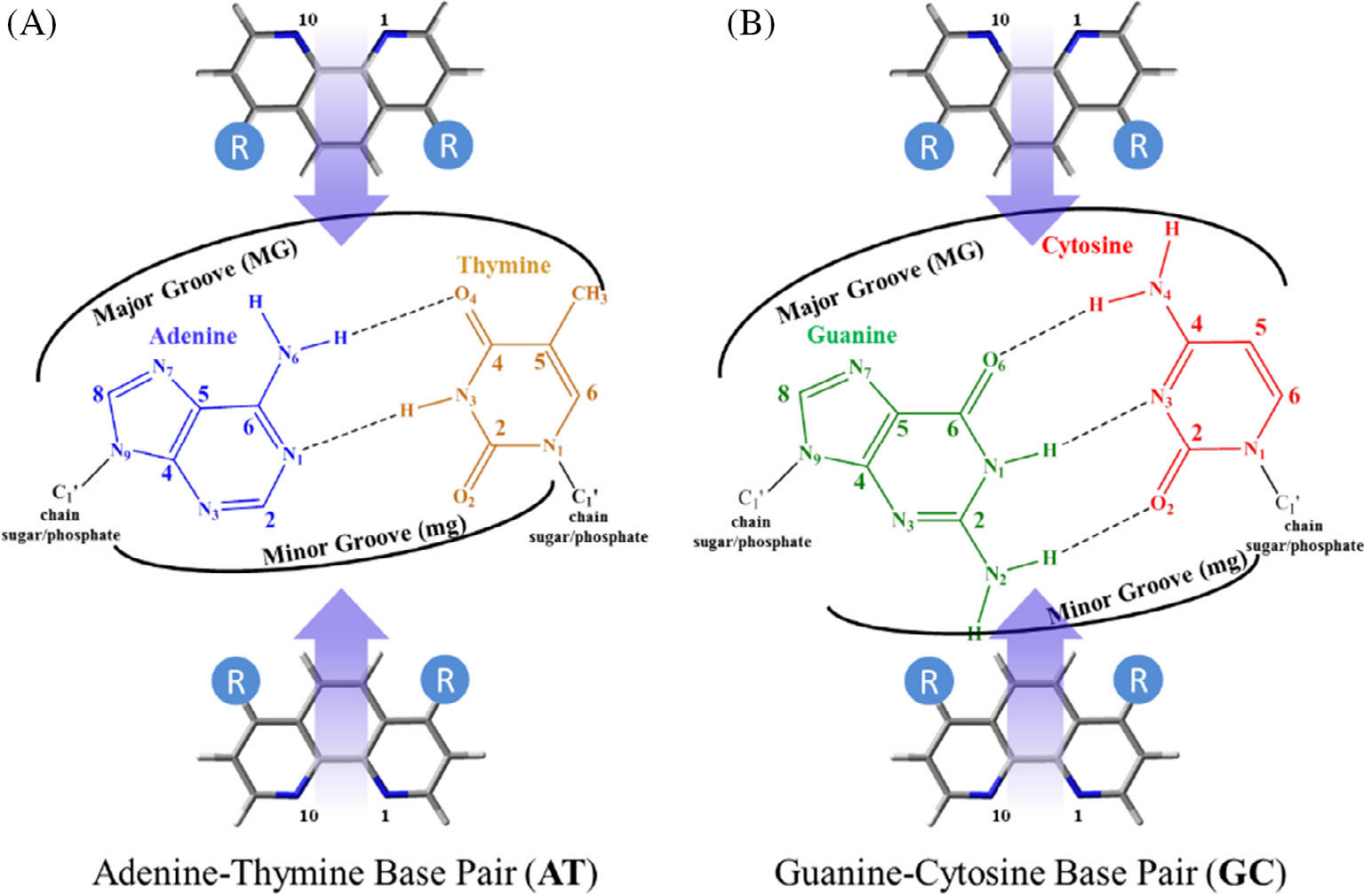
Scheme of AT (A) and GC (B) bps and the intercalated phen derivatives via minor groove (mg) and major groove (MG). R = H, NH_2_, or OH

We carried out semi‐empirical and DFT calculations including dispersion effects (see Computational Details) for different structures of adenine‐thymine/thymine‐adenine (AT/TA) and guanine‐cytosine/cytosine‐guanine (GC/CG) bps including the 4,7‐(OH)_2_phen, 4,7‐(NH_2_)_2_phen and phen intercalators by means of ring models.^56^ Moreover, we have also studied two orientations for the intercalation: through the mg and via MG (see Figure 2). Thus, in order to identify each system, the following nomenclature has been used along the work: (bps/intercalator/bps)groove (e.g., (GC/4,7‐(OH)_2_phen/CG)mg would be the system corresponding to the intercalation of 4,7‐(OH)_2_phen between GC/CG bps through the mg). In the following subsections, we shall discuss geometries and topological analysis of the studied structures, energetics, charge distribution and the evolution of the hydrogen bonded systems with time.

### 2.1 Geometries, topological QTAIM analysis of the electron density and NCI analysis

**FIGURE 3:**
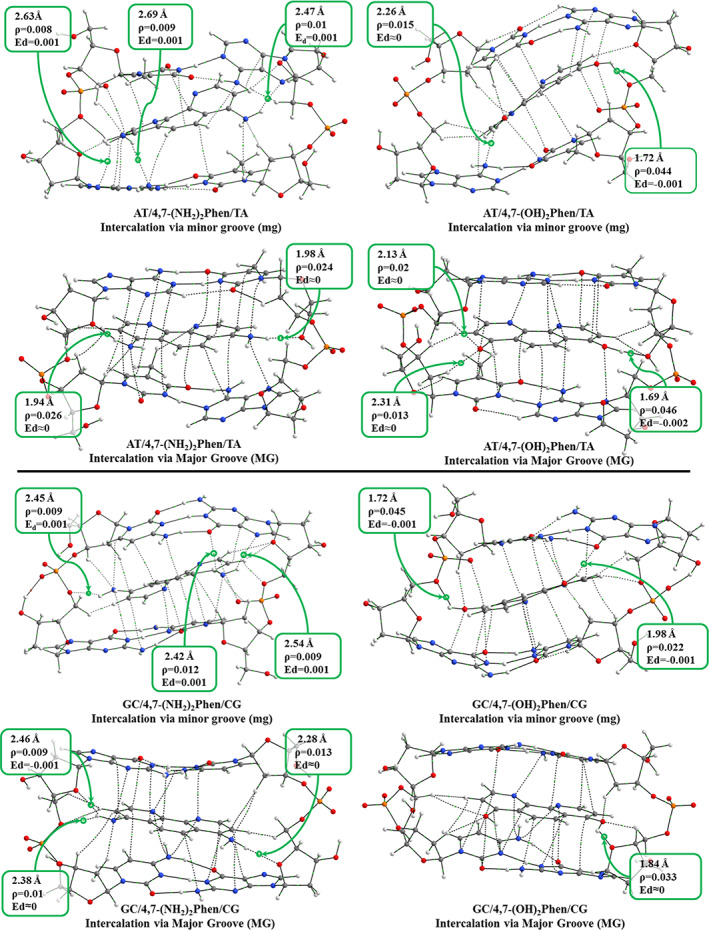
Bonding scheme from QTAIM topological analysis of the electron density (ρ) for intercalation of 4,7‐(NH_2_)_2_phen (left) and 4,7‐(OH)_2_phen (right) between AT/TA and GC/CG bps via mg and MG

The reader can find the cartesian coordinates of the optimized studied systems in the Supporting information. The identification of hydrogen bond interactions between the intercalators and bps was based on the QTAIM topological analysis of the electron density (ρ)^60^ bonding scheme and the obtained distances for such H‐bonds are shown in Figure 3 for the ring models of AT/X/TA and GC/X/CG systems via mg and MG. It must be said that a more complete QTAIM study of all the studied systems can be found in the Figures S2–S9. For each bond critical point (BCP), ρ value describes the strength of the interaction between two linked atoms, and the electron energy density (*E*_*d*_) describes the stability of the interaction, being values close to zero a characteristic for weak interactions like π‐π and H‐bonds. To show a clearer picture only bond paths between intercalator and DNA are represented (ring critical points, cage critical points, and intramolecular weak interactions in the sugar and phosphate backbone have been removed for clarity). For the represented intercalator‐ring model interactions, the bond paths where the functional groups —NH_2_ and —OH are involved are depicted with the corresponding values for the ρ, *E*_*d*_, and distances. Apart from the conventional hydrogen bonds involving the —NH_2_ and —OH functional groups, several π‐π, CH/π, CH/n and even H···H interactions have been found. Similar H···H interactions for nearby hydrogen atoms were already found in our previous works^30^, ^31^, ^33^, ^34^, ^35^, ^36^, ^61^ and corroborated for several kind of systems in the literature at different levels of theory.^62^, ^63^, ^64^ All these different weak interactions contribute to the interacting scheme in this kind of intercalating systems as we have reported in previous works.^30^, ^31^, ^33^, ^34^, ^35^, ^36^

It is observed that the distortion of geometries leads to a fewer number of π‐π interactions showing the most coplanar arrangements higher numbers of π‐π interactions. In both systems, GC/X/CG and AT/X/TA, the intercalation via mg yields more distorted geometries than the intercalation via MG, (the distortion is especially marked for the intercalation between AT/TA bps). It is observed that this distortion is due to the presence of interactions of —NH_2_ and —OH groups not only with atoms contained in the bps but also, and more important, with atoms of the sugar and phosphate backbone for at least one of the groups. For (AT/4,7‐(NH_2_)_2_phen/TA)mg system one of the —NH_2_ groups presents interactions with two heteroatoms of the nearby base pair, N7 and N6 of adenine (see Figure 3) located down‐left and the other —NH_2_ group shows only one interaction with an heteroatom of the nearby base, N7 of the adenine located up‐right, through a H‐bond. This situation is similar for (GC/4,7‐(NH_2_)_2_phen/CG)mg system where again a —NH_2_ group presents two interactions with atoms of the base pair, N7 and O6 of guanine up‐right (see Figure 3), whereas the other —NH_2_ group presents a H‐bond with the phosphate (left). For the intercalation of the 4,7‐(OH)_2_phen the general trend is that the —OH shows interactions with the atoms of the sugar and phosphate backbone although for the (AT/4,7‐(OH)_2_phen/TA)mg system one of the —OH functional groups still shows an interaction with the N6 atom of the adenine (down‐left), whereas for the (GC/4,7‐(OH)_2_phen/CG)mg system one —OH group presents a H‐bond with the O2 atom of the cytosine (up‐right). On the other hand, for the intercalation via MG, the H‐bond is formed with the sugar of the backbone (left).

**FIGURE 4:**
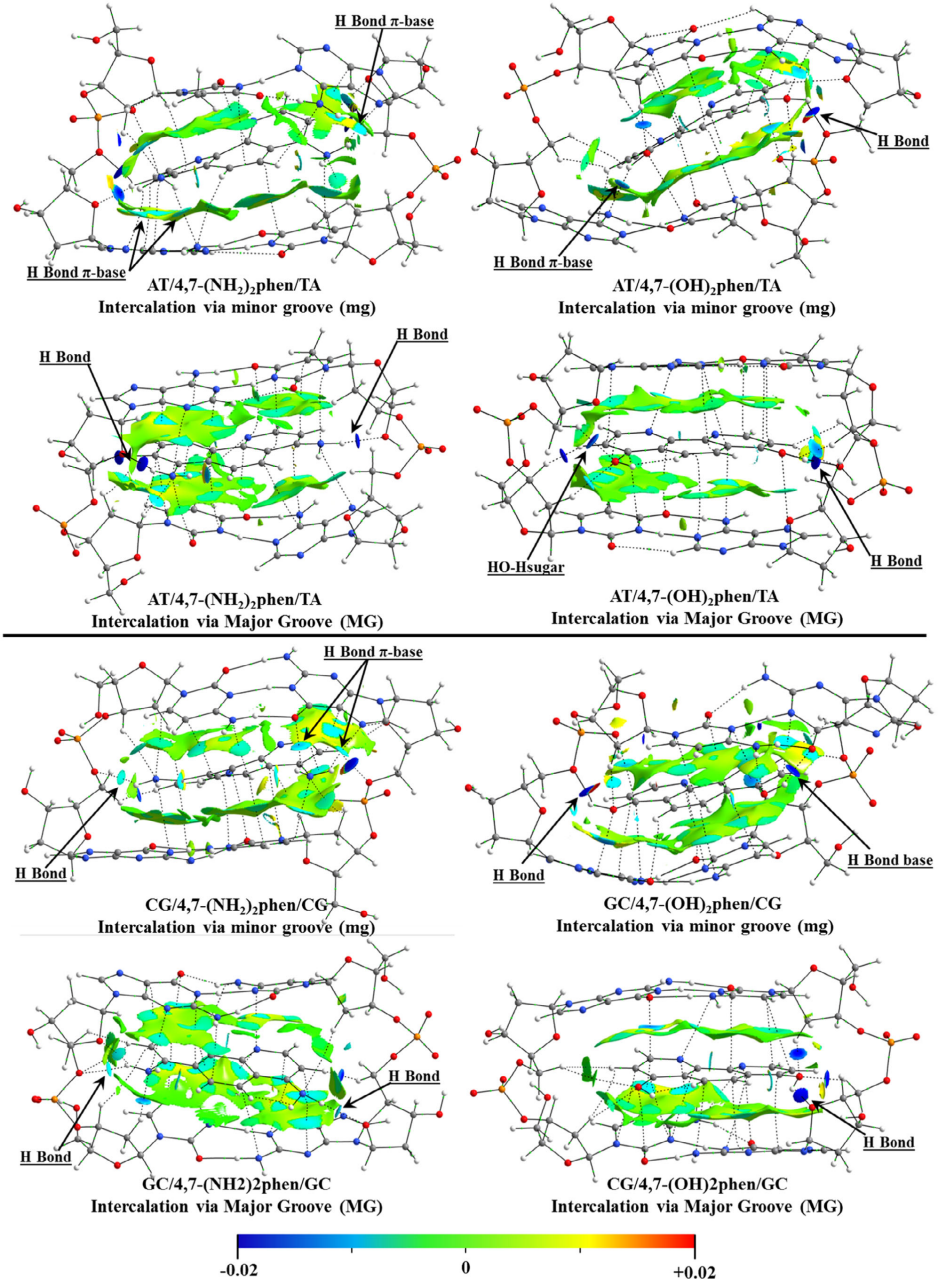
NCI index plots with gradient isosurfaces (*s* = 0.5 a.u.), colors according to values of sign(λ_2_)ρ, computed for the optimized structures of intercalation of 4,7‐(NH_2_)_2_phen (left) and 4,7‐(OH)_2_phen (right) between AT/TA and GC/CG bps via mg and MG

To gain insight on the bonding scheme between the intercalator and DNA, the NCI index^65^ plots are provided in Figure 4 for the same systems. NCI index displays low gradient (*s* = 0.5 a.u.) isosurfaces. The gradient isosurfaces are presented in a color map attending to the corresponding values of sign(λ_2_)ρ (second value of the hessian of ρ). Blue represents large negative value, indicative of attractive interactions, while red represents large and positive values indicative of non‐bonding interaction (steric crowding). Values close to zero correspond to van der Waals interactions, very weak interactions. The results showed from the NCI analysis indicate a good agreement with the QTAIM study where the most stabilized regions are located around the BCPs found from QTAIM.

For the AT/X/TA systems differences between the intercalation via mg and MG are observed. For the intercalation via mg we observe interactions of —NH_2_ and —OH with heteroatoms located in the plane of the bps, only for (AT/4,7‐(OH)_2_phen/TA)mg a formal hydrogen bond with an O atom of one of the phosphates (right) is presented for one of the —OH groups of the intercalator. For the intercalation via MG different results are shown, the main trend being the formation of hydrogen bonds between the substituents, —NH_2_ and —OH, with the O atoms present in the sugar and phosphate backbones. The most favorable intercalation appears for 4,7‐(NH_2_)_2_phen, in this case both —NH_2_ groups interact with conventional hydrogen bonds, with a strengthened attractive non‐covalent bonding interaction represented by large negative values of sign(λ_2_)ρ in blue, with the O atoms of both sugar and phosphate backbones. For the intercalation of 4,7‐(OH)_2_phen via MG, just one —OH group present a conventional hydrogen bond with the terminal O atom of the sugar and phosphate backbone, while the other O atom of the —OH group presents interactions with H atoms of the adjacent sugars (left).

Regarding the GC/X/CG systems, the intercalation via mg, yields interactions with the heteroatoms of the bps as we have observed before for AT/X/TA systems. For GC/4,7‐(NH_2_)_2_phen/CG we observe that one of the —NH_2_ groups yield a conventional hydrogen bond interaction with an O atom of the phosphate (left), while the other —NH_2_ group interacts mainly with heteroatoms of guanine, N7 and O6, (right‐up) with a considerable stabilizing interaction. This scheme is reproduced for the intercalation of GC/4,7‐(OH)_2_phen/CG via mg, where one of the —OH groups interact with an heteroatom of the base pair, O6 of cytosine (up‐right), and the other with an O atom of the sugar an phosphate backbone (left), in both cases these interactions are represented with a high negative value of (λ_2_)ρ for the NCI index. This is in agreement with the stabilization represented in the energy decomposition analysis (EDA) that will be observed below for the (GC/4,7‐(OH)_2_phen/CG)mg system where the stabilization by means of the electrostatic contribution is considerable. On the other hand, considering the (GC/4,7‐(NH_2_)_2_phen/CG)MG system, it is shown that one of the —NH_2_ groups presents two hydrogen bond interactions with O atoms of the sugar (left) while the other —NH_2_ group presents only one hydrogen bond with a sugar (right). For the (GC/4,7‐(OH)_2_phen/CG)MG system, it is observed that just one —OH group presents one hydrogen bond with a sugar (right), showing a high negative value for the NCI isosurface. Again, the intercalations via MG in the GC/CG systems yield interactions with the sugar and phosphate backbones avoiding the formation of interactions with the heteroatoms of the bps.

Summarizing, regarding the differences between the implementation of the —NH_2_ or —OH groups, the main difference is that the —OH tends to form more strengthened interactions (more negative value for the NCI index) with the O atoms present in the sugar and phosphate backbone. On the other hand, —NH_2_ mainly yields two interactions thorough hydrogen atoms with the heteroatoms of the bps. These interactions present a less negative value for (λ_2_)ρ indicating a weaker interaction in comparison to that presented by —OH groups. Only in the case of (AT/4,7‐(NH_2_)_2_phen/TA)MG and (GC/4,7‐(NH_2_)_2_phen/CG)MG systems the —NH_2_ groups interact only with O atoms of the sugar and phosphate backbone with very negative values of the NCI index.

### 2.2 Energies

The interaction energy Δ*E*_int_ between fragments can be split by the EDA into several contributions: the repulsive Δ*E*_Pauli_, which takes into account the destabilizing interactions between occupied orbitals, the electrostatic contribution, Δ*E*_elstat_, which is the classical electrostatic interaction between the unperturbed charge distributions of the rigid fragments and the orbital interaction contribution, Δ*E*_orb_, which accounts for the charge transfer and polarization contributions. Moreover, if an explicit correction term for dispersion interactions is employed, such extra term, Δ*E*_disp_, appears in the scheme.

(1) $$\Delta E_{\mathrm{int}}=\Delta E_{\text{Pauli}}+\Delta E_{\text{elstat}}+\Delta E_{\mathrm{orb}}\left(+\Delta E_{\text{disp}}\right).$$

**FIGURE 5:**
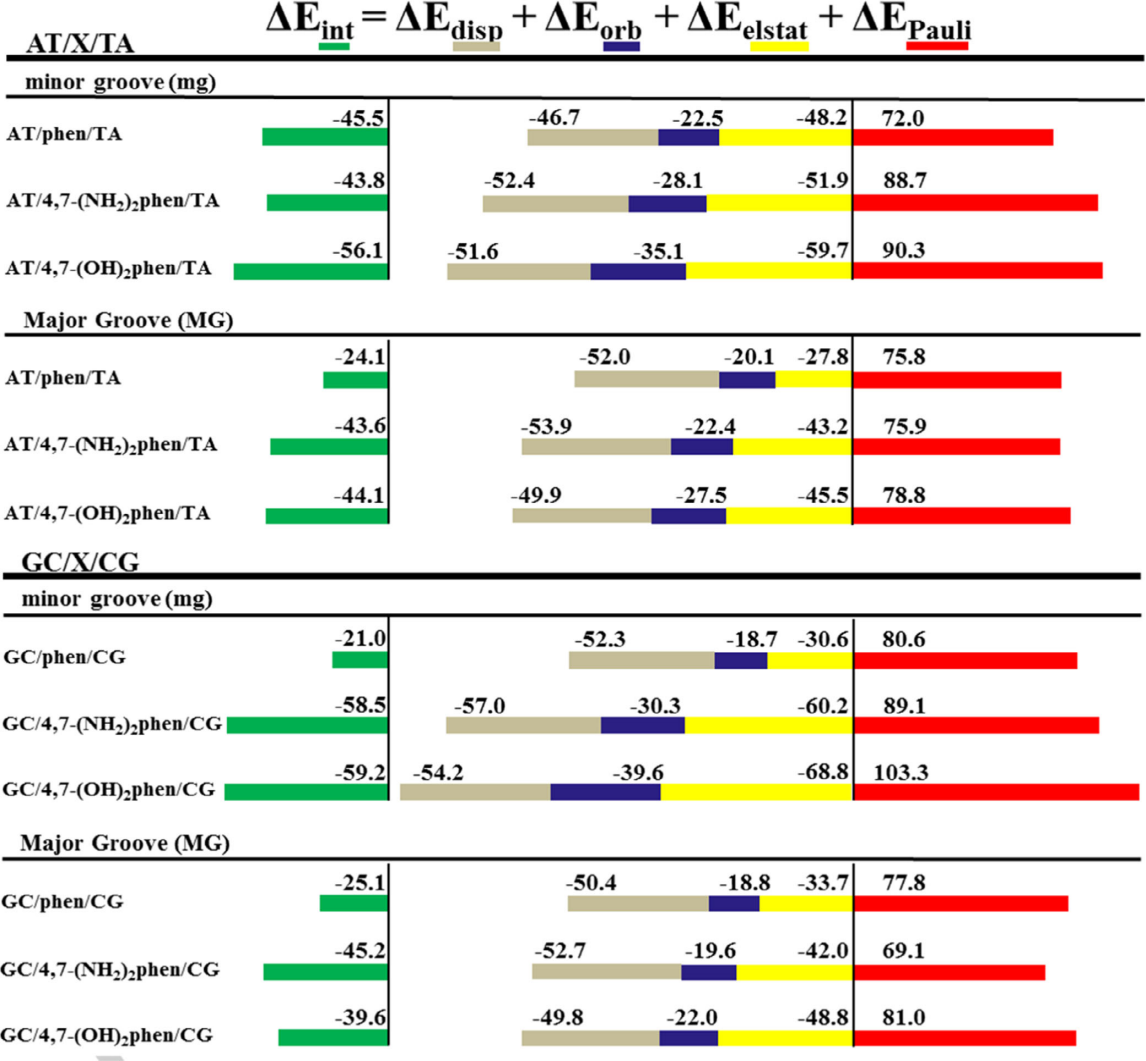
Cumulative bar diagram of the different contributions in the EDA at the B3LYP‐D3/TZP level (energy contributions in kcal mol^−1^)

Such EDA was carried out with the ADF software by means of the B3LYP‐D3 functional (see Computational Details) and the obtained results represented in a cumulative diagram for the intercalated systems are shown in Figure 5. Very interesting trends are observed for attractive and repulsive interactions that arise from the EDA analysis. First, attending to the repulsive contribution (Δ*E*_Pauli_) we observe the general trend for the intercalation via mg, where this repulsive contribution present higher values in comparison to the intercalation via MG, which could be attributed to repulsive interactions between the intercalator and the sugar and phosphate backbone, since in the intercalation via mg the intercalator is closer to sugar and phosphate backbone, whereas in the intercalation through the MG the sugar and phosphate backbone is farer away.

If we consider the attractive contributions, it must be highlighted that after substitution of phen with —OH and —NH_2_, the Δ*E*_elstat_ and Δ*E*_orb_ contributions are considerably increased for the intercalation via mg in GC/X/CG systems, being almost doubled regard the non‐substituted intercalator, while the Δ*E*_disp_ shows a discrete increase after the substitution for the intercalation through the mg. For the intercalation via MG the general trend is that the Δ*E*_elstat_ and Δ*E*_orb_ contributions present less pronounced increases. A remarkable behavior in these attractive contributions is that the Δ*E*_elstat_ is equal or even more important than the Δ*E*_disp_. This behavior not only differs from the behavior obtained for phen^30^, ^34^, ^36^ but also from the behavior of methylated phen^31^, ^33^, ^35^, ^36^ and ketonic derivatives of phen^32^, ^36^ where the Δ*E*_disp_ was the most important attractive interaction. Thus, the substitution of phen with —OH and —NH_2_ shows a very different behavior. Nevertheless, this is not surprising considering the dual nature of the forces ruling the hydrogen bond interactions: dispersion and electrostatic forces.^66^, ^67^ In our previous studies on the intercalation of phen^30^, ^34^, ^36^ and ketonic derivatives of phen^32^, ^36^ the main contribution to the interaction was the π‐π stacking, which is mainly ruled by dispersion forces. On the other hand, when phen was substituted with —CH_3_ ^31^, ^33^, ^35^, ^36^ CH/π, CH/n and even H···H interactions between the intercalators and DNA appeared. However, in these CH/π, CH/n, and H···H interactions the dispersive nature of such weak interactions rules the interaction, whereas the electrostatic compound plays minor role.^68^, ^69^, ^70^, ^71^, ^72^, ^73^, ^74^, ^75^, ^76^, ^77^, ^78^, ^79^, ^80^, ^81^ Now, having —OH and —NH_2_ functional groups in the phen intercalator, conventional hydrogen bonds (between hard acids and hard bases) appear in which the electrostatic forces play a major role in the interaction^82^, ^83^ and it is reflected in the EDA with an increase of the Δ*E*_elstat_ contribution. On the other hand, we observe another characteristic behavior for these systems with —OH and —NH_2_ in the Δ*E*_orb_ contribution, which becomes more important than for the unsubstituted phen. Because Δ*E*_orb_ is associated to polarization and charge transfer processes, these more negative values of Δ*E*_orb_ for the systems with —OH and —NH_2_ can be attributed again to the conventional hydrogen bonds formed between the 4,7‐(OH)_2_phen and 4,7‐(NH_2_)_2_phen ligands and the DNA, in which a more important charge transfer is produced between the intercalator and the DNA by means of these conventional hydrogen bonds as we will see below.

Taking into account all the contributions, in general, the total interaction energy (Δ*E*_int_) gets reinforced after the substitution with —NH_2_ and —OH groups, being the most stabilized systems the (GC/4,7‐(NH_2_)_2_phen/CG)mg and (GC/4,7‐(OH)_2_phen/CG)mg. These results for 4,7‐(NH_2_)_2_phen and 4,7‐(OH)_2_phen intercalating via mg could be explained with the hydrogen bonds and BCPs found at the same time with the sugar and phosphate backbone and the heteroatoms of the bps (see Figures 3 and 4), where the H atoms of the functional groups form BCPs with the O atoms of the sugar and phosphate backbone while the N and O atoms form BCPs with atoms of the bps.

Even though DFT‐D methods contains corrections to dispersion contributions, since there are several functionals and different kinds of corrections to dispersion, one could think about some validation of the Δ*E*_int_ in the EDA. Of course, it is not possible to compare the Δ*E*_int_ from the EDA with any experimental affinity by using the intercalation through ring models of DNA that we have used in our study. Nevertheless, it is possible to use benchmark calculations with highly correlated methods as CCSD(T) with extended basis sets to have an idea of the accuracy of the Δ*E*_int_ obtained through DFT‐D. Actually, after the consolidation of conventional DFT approximations, different studies were reported where a G2 modified composite methodology in which MP2 geometries and HF frequencies were substituted by the DFT ones and the QCISD(T) calculations were replaced by CCSD(T) ones.^84^, ^85^ It must be said that we already performed similar calculations by considering single‐point calculations through highly correlated CCSD(T) on DFT optimized geometries with systems of few tens of atoms^86^, ^87^, ^88^, ^89^ because of the highly demanding resources of such kind of computations in terms of time, memory and disk space. These highly correlated methods were restricted to systems with a reduced number of atoms 20 years ago. Nevertheless, taking the advantage of the recent and efficient developed algorithm for CCSD(T) calculations, the domain‐based local pair natural orbital approach (DLPNO‐CCSD(T)),^90^, ^91^ which is less computationally‐demanding, we were able to perform CCSD(T) with ~150 atoms with our local cluster resources for benchmark energy calculations as we already did in a previous study^92^ in which we calculated accurate interaction energies between G‐tetrads and alkaline atoms of DNA G‐quadruplexes. In addition, such methodology was also proven useful in previous computational studies^93^ where interaction energies in supramolecular flat complexes, with large aromatic moieties, where mainly ruled by weak interactions like π‐π stacking and CH/π interactions, also present in the intercalation of flat ligands between DNA bps.

**FIGURE 6:**
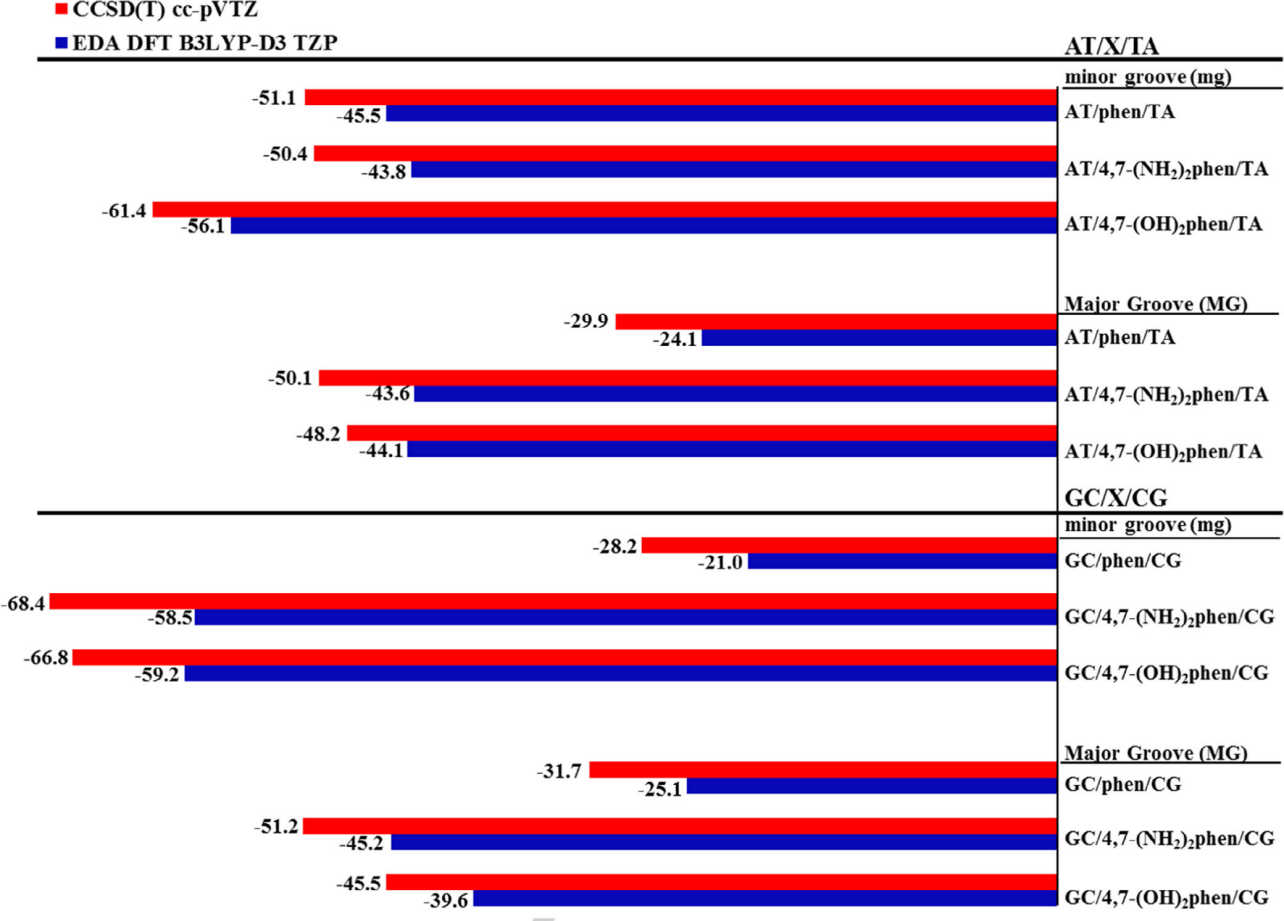
Bar diagram for the interaction energies of the intercalation processes obtained at CCSD(T)/cc‐pVTZ level of theory (red) and the interaction energy obtained from the EDA analysis at B3LYP‐D3/TZP level

Thus, in order to obtain accurate benchmark results regarding the interaction energy between the considered fragments, intercalator and ring model of DNA, DLPNO‐CCSD(T) calculations were performed to corroborate the accuracy of the interaction energies obtained from the EDA results shown above. Figure 6 shows such comparison between the interaction energies obtained with the EDA at DFT‐D level and the DLPNO‐CCSD(T) methodology.

According to the results obtained from the interaction energy between the considered fragments, computed at DLPNO‐CCSD(T) level, we observe that the trends obtained from the EDA analysis matches nicely with the accurate energies provided by the accurate benchmark DLPNO‐CCSD(T) results. In consequence, we may assume that the relative interaction energies between the substituted phen derivatives obtained by DFT‐D methods, and the contributions depicted above, are reliable.

To gain insight about the effect of the solvent in the intercalation process, the contribution of the desolvation penalty, Δ*E*_Solv_, was considered and added to the total interaction energy, Δ*E*_int_, from EDA to obtain the final energy in solution, Δ*E*_aq_ (see Tables 1 and 2).

**TABLE 1:** Contributions of the solvation energies (kcal mol^−1^) for the (AT/X/TA) intercalated systems at the B3LYP‐D3/TZP level by using COSMO approach

AT/X/TA			
	mg		
	phen	4,7‐(NH_2_)_2_phen	4,7‐(OH)_2_phen
*E*_Solv_ (total system)	−94.8	−101.5	−100.0
*E*_Solv_ (intercalator)	−15.0	−28.0	−25.4
*E*_Solv_ (pocket)	−100.2	−100.1	−105.0
Δ*E*_Solv_ ^a^	20.4	26.6	30.4
Δ*E*_aq_ ^b^	−25.1	−17.1	−25.7

	MG		
	phen	4,7‐(NH_2_)_2_phen	4,7‐(OH)_2_phen
*E*_Solv_ (total system)	−103.2	−106.7	−108.4
*E*_Solv_ (intercalator)	−15.1	−29.0	−24.7
*E*_Solv_ (pocket)	−99.2	−103.4	−102.2
Δ*E*_Solv_ ^a^	11.1	25.7	18.5
Δ*E*_aq_ ^b^	−13.0	17.9	−25.6

a Δ*E*_Solv_ = *E*_Solv_ (total system) − *E*_Solv_ (intercalator) − *E*_Solv_ (pocket).

b Δ*E*_aq_ = Δ*E*_Int_ + Δ*E*_Solv_.

**TABLE 2:** Contributions of the solvation energies (kcal mol^−1^) for the (GC/X/CG) intercalated systems at the B3LYP‐D3/TZP level by using COSMO approach

GC/X/CG			
	mg		
	phen	4,7‐(NH_2_)_2_phen	4,7‐(OH)_2_phen
*E*_Solv_ (total system)	−123.6	−105.5	−99.1
*E*_Solv_ (intercalator)	−15.6	−27.9	−25.4
*E*_Solv_ (pocket)	−116.6	−107.1	−108.7
Δ*E*_Solv_ ^a^	8.6	29.5	35.0
Δ*E*_aq_ ^b^	−12.5	−29.0	−24.2

	MG		
	phen	4,7‐(NH_2_)_2_phen	4,7‐(OH)_2_phen
*E*_Solv_ (total system)	−108.7	−116.0	−113.8
*E*_Solv_ (intercalator)	−15.6	−28.0	−25.1
*E*_Solv_ (pocket)	−104.3	−120.1	−112.8
Δ*E*_Solv_ ^a^	11.2	32.1	24.1
Δ*E*_aq_ ^b^	−13.9	−13.1	−15.5

^a^ Δ*E*_Solv_ = *E*_Solv_ (total system) − *E*_Solv_ (intercalator) − *E*_Solv_ (pocket).

^b^ Δ*E*_aq_ = Δ*E*_Int_ + Δ*E*_Solv_.

We observe that for the AT/X/TA systems (Table 1) the most stabilized structures after desolvation are (AT/4,7‐(OH)_2_phen/TA)mg (−25.7 kcal mol^−1^) for the mg and (AT/4,7‐(OH)_2_phen/TA)MG (−25.6 kcal mol^−1^) for the MG. Such systems were also the most stable systems when solvent effects were not taken into account and actually, the general trends in stabilization are maintained when comparing the systems with and without solvent effects. Thus, for the AT/X/TA systems, even the Δ*E*_Solv_ penalty changes the final energy of the systems; the trends in stabilization are maintained. Moreover, we observe that the Δ*E*_Solv_ penalties are more important for the systems intercalating via mg. Regarding the GC/CG systems the most stabilized systems considering the solvent effects are (GC/4,7‐(NH_2_)_2_phen/CG)mg with a final energy of −29.0 kcal mol^−1^ for the mg and (GC/4,7‐(OH)_2_phen/CG)MG for which the final energy after considering desolvation penalty is −15.5 kcal mol^−1^ for the MG. In this case the trends when taking into account solvent effects are not the same than without consideration of solvent effects (see Figure 5). It means that even though the Δ*E*_elstat_ achieves an important role, which is reflected in the Δ*E*_int_, for these intercalators forming conventional hydrogen bonds, the solvent effects by means of the Δ*E*_Solv_ penalty could change the final trends. Therefore, the main conclusion arising from the intercalation models is that we also have to keep in mind that the inclusion of solvent effects may change the stability of the systems. This last point would be in agreement with the different experimental results obtained for intercalating crystal structures versus intercalation processes in solution.^8^, ^21^

### 2.3 Analysis of the charge distribution

We can go one step beyond on the analysis of the electronic structure with the information extracted from charge distribution of the studied systems. Table 3 shows, for the studied systems, the charge distribution among the different fragments of the structures: the intercalator, each DNA base and each sugar and phosphate backbone including the sodium cations.

**TABLE 3:** Distribution of the Hirshfeld charges on the intercalators, DNA bases and sugar and phosphate backbone including sodium cations

Structures	Q_Backbone_	Q_Purine_	Q_Pyrimidine_	Q_Intercalator_
(AT/4,7‐(NH_2_)_2_phen/TA)mg	−0.037/0.312	−0.006/0.087	−0.093/−0.191	0.037
(AT/4,7‐(OH)_2_phen/TA)mg	0.057/0.234	0.008/−0.051	−0.099/−0.199	0.049
(AT/4,7‐(NH_2_)_2_phen/TA)MG	0.181/0.313	−0.042/−0.078	−0.169/−0.134	−0.071
(AT/4,7‐(OH)_2_phen/TA)MG	0.208/0.369	−0.056/−0.078	−0.163/−0.212	−0.059
(GC/4,7‐(NH_2_)_2_phen/CG)mg	0.249/−0.059	−0.191/−0.208	0.014/0.071	0.122
(GC/4,7‐(OH)_2_phen/CG)mg	0.041/0.221	−0.153/−0.237	0.091/0.004	0.032
(GC/4,7‐(NH_2_)_2_phen/CG)MG	0.256/0.233	−0.229/−0.241	−0.004/0.021	−0.035
(GC/4,7‐(OH)_2_phen/CG)MG	0.251/0.218	−0.232/−0.213	−0.032/0.019	−0.012

Looking at the charge values on the intercalators of all the analyzed systems, which would correspond to the charge transfer between the DNA pocket and the intercalator, there is not a significant charge transfer as a general trend with the exception of the (GC/4,7‐(NH_2_)_2_phen/CG)mg system. It means that, even we have strong conventional hydrogen bonds in the studied systems, the charge transfer between DNA and the intercalator will have minor role in the interaction. This behavior is in agreement with the Δ*E*_orb_ numbers of the EDA observed in the previous section for which the highest value was for the (GC/4,7‐(NH_2_)_2_phen/CG)mg structure although the Δ*E*_orb_ contribution is always the lowest attractive term when comparing to Δ*E*_elstat_ or Δ*E*_disp_. On the other hand, an interesting behavior is observed for the sign of the charge of the intercalator. That is, the sign depends on the orientation of the intercalation process more than on the nature of the species involved in the process and for the systems intercalating through the mg the sign of the charge located on the intercalator is positive, whereas for the systems intercalating via MG the sign is negative. We would like to highlight this result because it breaks the classical behavior found in the bibliography for the studies of intercalators at QM level. Indeed, in the pioneer studies of Rěha et al.^46^ the intercalators were described as good π‐electron acceptors based on the analysis of their frontier molecular orbitals (HOMO and LUMO) in their three‐body models. They studied ethidium, daunomycin, ellipticine and 4,6′‐diaminide‐2‐phenylindole on AT and GC bps and their models used for calculations were very flat and had a very good overlap between intercalator and bps. Subsequent studies by El‐Gogary et al.^48^, ^49^ also used these three‐body models of coplanar structures, which were optimized at different levels of calculation and they also found that, based on the frontier orbitals analysis, the intercalators acted as electron acceptors. Even in our more recent studies on the intercalation of phen and phen derivatives^30^, ^32^, ^36^ by using sandwich models^56^ without sugar and phosphate backbone we found that the intercalator was negatively charged independently of the orientation (intercalation through the mg or via MG) in the different studied systems indicating low charge transfer to the intercalators and therefore acting as electron acceptor even though that this charge transfer contribution was low and its role was not the most important in the interaction as observed in the Δ*E*_orb_ of the respective EDAs. Thus, when incorporating —NH_2_ and —OH groups to phen, which may form conventional hydrogen bonds, in the most sophisticated ring model including sugar and phosphate backbone, the addition of these new fragments along with the addition of the —NH_2_ and —OH groups could perturbate the final system in such a way that depending on the orientation of the intercalation, we would be able to reverse the sense of polarization we have in these biological processes. In these ring models with the 4,7‐(OH)_2_phen and 4,7‐(NH_2_)_2_phen intercalators the classical behavior of the intercalator as π‐electron acceptor occurs when the intercalation takes place through the MG where the in intercalator and bps remains almost coplanar and we find a high number of BCPs corresponding to π‐π interactions (see Supporting information). On the other hand, when the intercalation takes place via mg we observe a more distorted structure for the intercalator (see Figure 3) and also the number or BCPs corresponding to π‐π interactions have decreased (see Supporting information). Thus, we believe that the lower overlap between the intercalator and bps produced because of the distortion of the planarity of the intercalator when intercalates through the mg and the perturbation that the sugar and phosphate backbone may produce to the electronic properties of the system, when comparing to the seminal three‐body model and sandwich models, could explain this change of sign of the charge transfer between the DNA pocket and the intercalator being such charge transfer in any case very small.

### 2.4 MD simulations and analysis of the evolution of the hydrogen‐bonded systems with time

In order to see the evolution of the intercalation of the phen derivatives with time, MD simulations were performed. Because this work is based on QM methods, to preserve a similar computational level, semi‐empirical MD simulations have been considered with the same PM6‐DH2 Hamiltonian.

**FIGURE 7:**
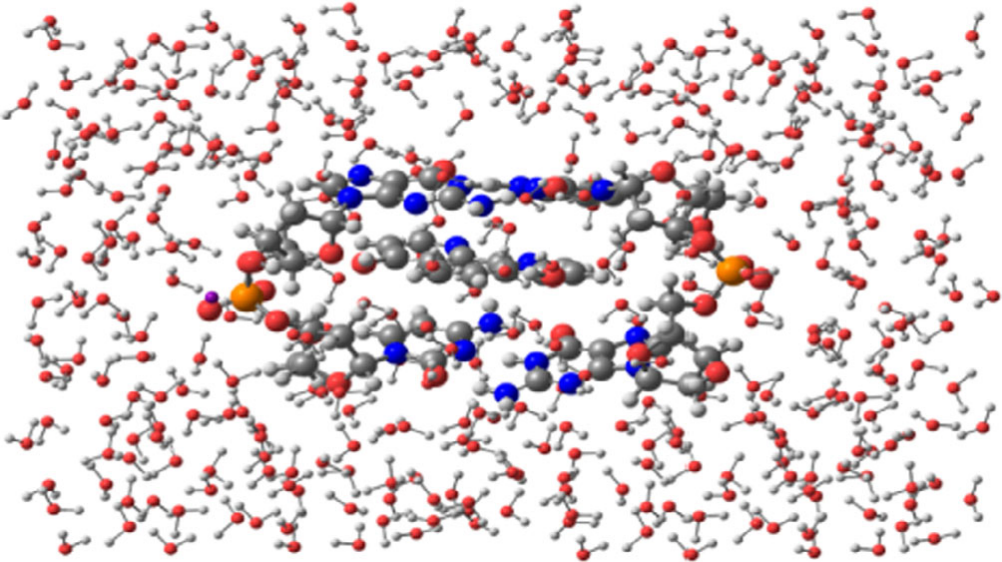
(GC/4,7‐(OH)_2_phen/CG)MG system inside a box of explicit water molecules taking into account the TIP3PBOX solvent model in a rectangular box with edges no closer than 5 Å to any atom of the (GC/4,7‐(OH)_2_phen/CG)MG structure

**FIGURE 8:**
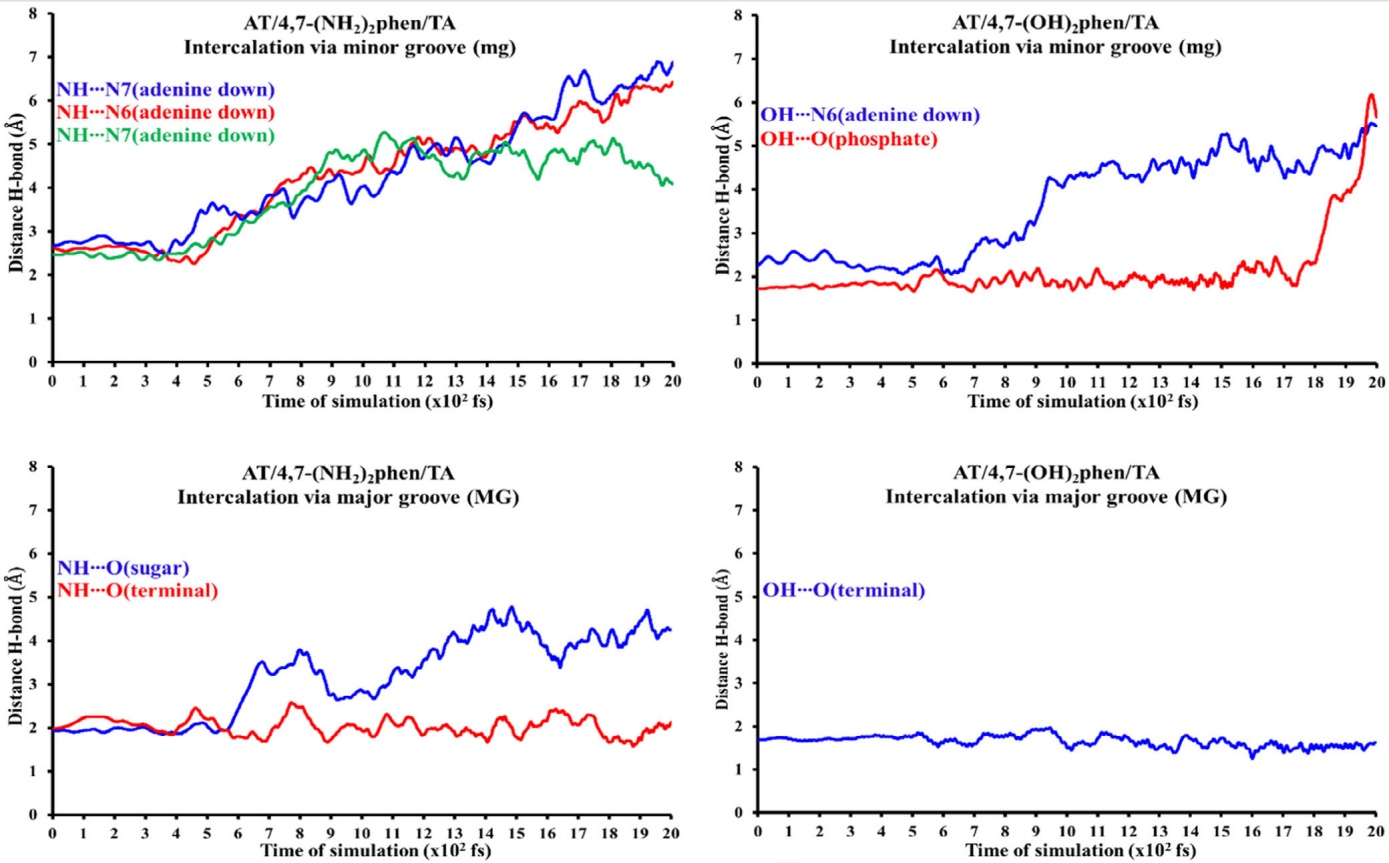
Monitoring of the evolution for the different conventional hydrogen bonds formed in the AT/X/TA systems. Distances in Å and time of MD simulation in fs (total MD simulation time at PM6‐DH2 level: 2 ps)

**FIGURE 9:**
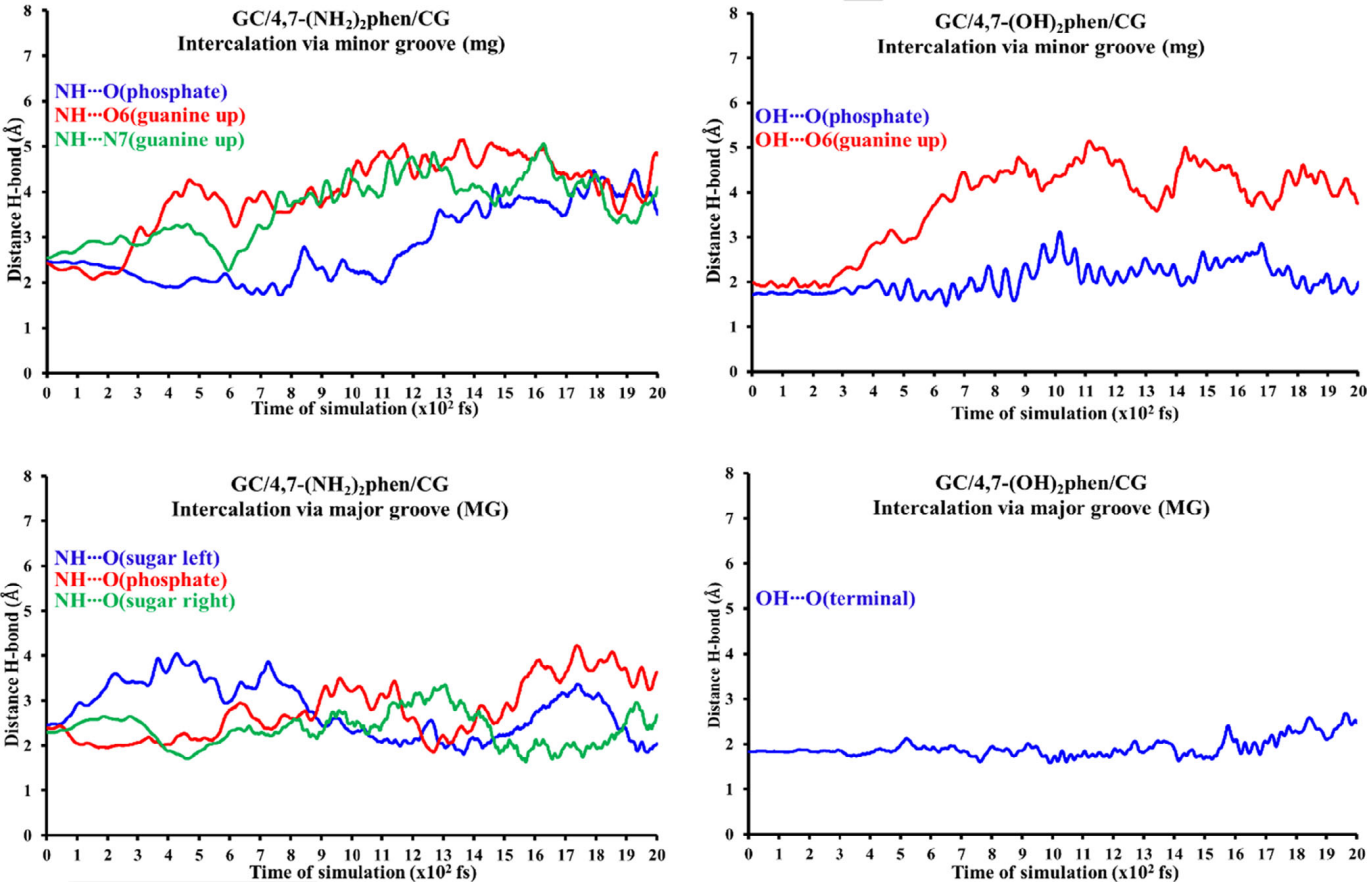
Monitoring of the evolution for the different conventional hydrogen bonds formed in the GC/X/CG systems. Distances in Å and time of MD simulation in fs (total MD simulation time at PM6‐DH2 level: 2 ps)

The stability of the conventional hydrogen bonds found in our systems have been monitored by means of semi‐empirical MD simulations until 2 ps. This range of time in the simulation was chosen because bond vibrations and some chemical reactions can be achieved in the range scale between fs and ps.^94^, ^95^, ^96^ Therefore, simulations of 2 ps would be enough to check the stability of our hydrogen bonded systems, which are weaker than covalent bonds involved in chemical reactions. Steps of 1 fs were taken into account for the MD simulations. On the other hand, the studied systems were introduced into a box of explicit water molecules. The TIP3PBOX solvent model was considered by using a rectangular box with edges no closer than 5 Å to any atom of the studied ring models (see Figure 7 as an example for the representation of the structures and see Supporting information for the movies of all MD simulations). Figures 8 and 9 show the evolution of the different conventional hydrogen bonds formed in the AT/X/TA and GC/X/CG systems, respectively.

As a general trend all the conventional hydrogen bonds formed with any of the —NH_2_ and —OH functional groups with the heteroatoms of the bps, mainly O and N atoms, tend to disappear between 400 and 900 fs. On the other hand, the conventional hydrogen bonds formed with the sugar‐phosphate backbone have different behavior depending on the substitution (—NH_2_ or —OH). The bonds formed with —OH remain stable during all the time of simulation (2 ps). Nevertheless, it must be said that when the systems arrive at this threshold an important distortion of the ring model and the entry of some water molecules into the intercalation pocket was found as a general trend for the studied ring model systems (see MD movies in the Supporting information), which suggests that the ring model is a quite good approach for non‐dynamic studies but when a box of water molecules is included in MD simulations the ring model presents some shortcomings due to the lack of the stabilization that would be produced if more stacked bps were considered in the tetramer, hexamer, octamer, decamer, dodecamer, or any other larger duplex DNA model. On the other hand, specially interesting is the behavior of the conventional hydrogen bonds formed between the —NH_2_ and the O atoms of the sugar and phosphate backbone.

In the case of the (AT/4,7‐(NH_2_)_2_phen/TA)MG system, for which we have a NH···O(Terminal) hydrogen bond, this interaction is maintained during all the time of the MD simulation (2 ps). However, in the same system we find a NH···O(sugar) hydrogen bond that disappear about 600 fs. In the case of the (GC/4,7‐(NH_2_)_2_phen/CG)mg system, for which we find a NH···O(phosphate) hydrogen bond, this interaction disappear about 1.1 ps. Finally, for the (GC/4,7‐(NH_2_)_2_phen/CG)MG system, in which we have also a NH···O(phosphate) hydrogen bond, it disappears also about 1.3 ps. In this last (GC/4,7‐(NH_2_)_2_phen/CG)MG structure we have also two NH···O(Sugar) interactions as for the (AT/4,7‐(NH_2_)_2_phen/TA)MG system. Nevertheless, now, and different from the (AT/4,7‐(NH_2_)_2_phen/TA)MG structure, such interaction remain during the 2 ps of the MD simulation. Interesting is the case of the NH···O(sugar left), which have very width fluctuations from 1.81 to 4.04 Å but at the end of the 2 ps MD simulation, the NH···O(sugar left) hydrogen bond distance is still 2.04 Å and therefore it is maintained during all the simulation time. All these trends in the MD simulation would be in agreement with the values of the ρ in the BCPs of the QTAIM analysis and the values of the isosurfaces of the NCI analysis corresponding to the monitored conventional hydrogen bonds since the values of ρ and such isosurfaces are higher for the conventional hydrogen bonds coming from the —OH than for those produced by the —NH_2_ functional groups. On the other hand, since the —OH group only have one H atom to form the hydrogen bonds, whereas the —NH_2_ group has two H atoms, competence could be produced in the latter case and more possibilities to form hydrogen bonds with the explicit water molecules are expected. These could be the reasons, along with the distortion of the ring models during the MD simulation, for the irregular behavior of the systems containing 4,7‐(NH_2_)_2_phen. Moreover, these results show not only the important role of the sugar and phosphate backbone to describe properly the intercalation of 4,7‐(OH)_2_phen and 4,7‐(NH_2_)_2_phen between DNA bps but also the limitations of the ring model, in which the lack of the stabilization produced by the rest of the stacked bps leads to the distortion of the system during the MD simulation (see movies in the Supporting information).

**FIGURE 10:**
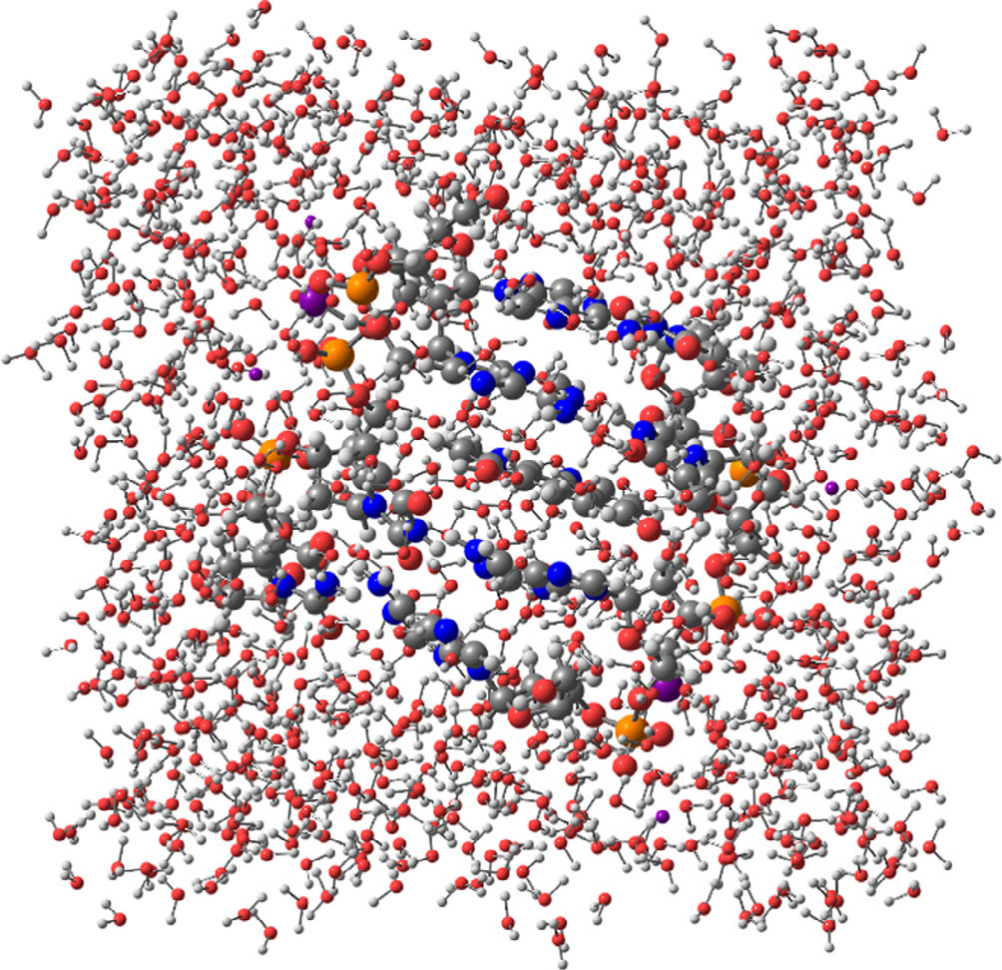
Model system for the d(AATT)_2_ tetramer intercalating the4,7‐(OH)_2_phen ligand between AT/TA bps through the mg inside a box of explicit water molecules taking into account the TIP3PBOX solvent model in a rectangular box with edges no closer than 5 Å to any atom of the d(AATT)_2_ tetramer intercalating the4,7‐(OH)_2_phen ligand via mg

As observed before at the end of the simulations (see movies in the Supporting information) the inclusion of H_2_O molecules between the H bonds of bps leads to the total distortion of the ring model. In order to shed light in the evolution of the H‐bonds for an intercalated system between DNA bps with a more realistic DNA structure, we considered a DNA tetramer where the corresponding π‐π interactions between the stacked bps are present, which contribute to keep the stacked DNA arrangement. We have chosen the larger d(AATT)_2_ tetramer model where a 4,7‐(OH)_2_phen ligand was intercalated via mg in order to observe the evolution of the conventional hydrogen bonds with a more realistic model. The intercalation between the bps is according to the methodology explained below and similar MD simulation conditions has been performed as for the ring model. In Figure 10 the geometrical arrangement of the d(AATT)_2_ intercalating the 4,7‐(OH)_2_phen ligand between AT/TA bps via mg is shown along with the corresponding solvation environment.

Performing again MD simulations until 2 ps, we observed that for the chosen larger system including the d(AATT)_2_ DNA tetramer and the intercalated 4,7‐(OH)_2_phen ligand via mg, the whole structure keeps the geometrical arrangement along all the time of simulation. The main conclusion that arises from this MD simulation is that the π‐π stacking of the successive bps avoid the deformation observed above with the smaller ring systems where the solvent molecules interacted with the hydrogen bonds of the confronted bps.

**FIGURE 11:**
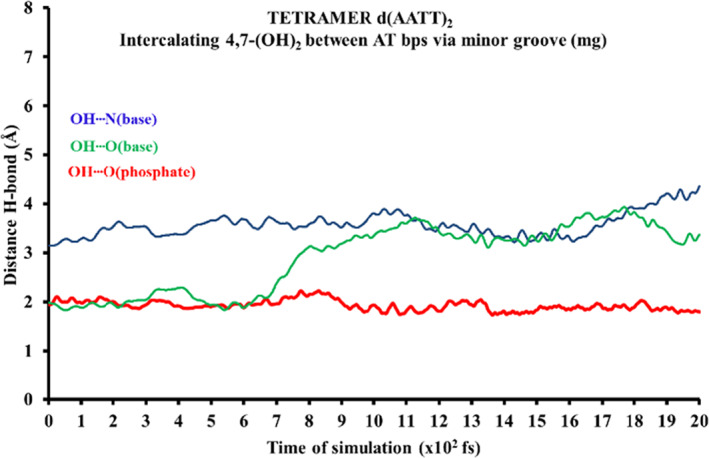
Monitoring of the evolution for the different conventional hydrogen bonds formed in the d(AATT)_2_ tetramer intercalating 4,7‐(OH)_2_phen between AT bps through the mg. Distances are in Å and time of MD simulation in fs (total MD simulation time at PM6‐DH2 level: 2 ps)

Attending to the evolution of the hydrogen bonds for the intercalated4,7‐(OH)_2_phen in the tetramer DNA structure via mg, we observe that the hydrogen bond formed with the phosphate group remains unchanged along all the simulation (see Figure 11 and movie in the Supporting information **)**. On the other hand, the other OH group that is located between the two bps presents a different behavior. After 7 ps the distance with the hydrogen bond formed with one O atom of a nearby base is increased and after this point this H atom remains equidistant between the two bps with a slight fluctuation of the distances. The main conclusion that arises from the MD simulations is that the conventional hydrogen bonds produced by the studied phen derivatives with the sugar and phosphate backbone tend to be stable with time, whereas those with the bps tend to disappear at some point of the MD simulation. On the other hand, after comparison of the MD simulation for the intercalated system in the d(AATT)_2_ DNA tetramer and the intercalated systems with the ring models, it is mandatory to use at least the DNA tetramer for MD simulations, which produces the stabilization of the system because of the stacking forces of the additional bps compared to the ring model, which becomes distorted at the end of the MD simulations because of the lack of such stacking stabilization that would be produced by the addition of the two more bps in the DNA tetramer.

## 3 CONCLUSION

In order to analyze the effect of hydrogen bonds of phen derivatives in the structure, energetics, interaction properties and their evolution in time, optimizations and MD simulations were carried out on GC/CG and AT/TA ring model systems incorporating phen derivatives as intercalators, which were made from substitution of ‐H atoms in positions 4 and 7 by the —OH and —NH_2_ functional groups (4,7‐(OH)_2_phen and 4,7‐(NH_2_)_2_phen). We used semi‐empirical and DFT methods both including dispersion effects to analyze this kind of systems in which dispersion forces are crucial. When these phen derivatives intercalate between DNA bps the —OH and —NH_2_ groups are able to form hydrogen bonds not only with several atoms of the bps but also with atoms of the sugar and phosphate backbone, which help to the stabilization of this kind of ligands during the intercalation process with the eventual improvement of their cytotoxicity. We considered two possibilities for the orientation of the intercalation: (1) through the mg and (2) via MG.

After the study of the geometries, QTAIM topologies, NCI analysis, energetics, charges and the evolution of the systems with time by means of 2 ps MD simulations in a box of explicit water molecules, we concluded that the sugar and phosphate backbone plays a crucial role in the stabilization of the phen derivatives containing —NH_2_ and —OH functional groups. This is mainly because the systems studied in this work, 4,7‐(OH)_2_phen and 4,7‐(NH_2_)_2_phen, are able to form hydrogen bonds not only with the heteroatoms of the surrounding bps of DNA but also with the O atoms of the sugar and phosphate backbone, the latter more stable in time. Such interactions with the backbone results of high importance in the behavior of the intercalator and its eventual cytotoxicity.

On the other hand, the information given by the EDA provides also very interesting conclusions. First of all, the intercalation of 4,7‐(OH)_2_phen and 4,7‐(NH_2_)_2_phen ligands through the mg leads to a more negative Δ*E*_int_ than their counterparts intercalating via MG as a general trend. This is because the intercalation through the mg is able to produce more conventional hydrogen bonds not only with the bps but also with the sugar and phosphate backbone than when intercalation is produced via MG. The second important conclusion derived from the EDA is that, different from our previous studies^30^, ^31^, ^32^, ^33^, ^34^, ^35^, ^36^ considering phen and methyl or ketonic derivatives of phen in which the Δ*E*_disp_ was the most important attractive contribution to the interaction energy, now the Δ*E*_elstat_ attractive contribution is equal or even a little bit more important than Δ*E*_disp_. This is not surprising considering that now 4,7‐(OH)_2_phen and 4,7‐(NH_2_)_2_phen ligands with —OH and —NH_2_ functional groups are able to form conventional hydrogen bonds not only with the bps but also with the sugar and phosphate backbone and the electrostatic contribution in conventional hydrogen bonds results much more important than weaker CH/n, CH/π or H···H hydrogen bonds for which the dispersion contribution was dominant. The last remark from the EDA is that the Δ*E*_orb_ accounting for charge transfer and polarization effects is more important for 4,7‐(OH)_2_phen and 4,7‐(NH_2_)_2_phen ligands than for phen, which is related again to the —OH and —NH_2_ functional groups that may form conventional hydrogen bonds and such interactions allow higher charge transfer as can be confirmed by the values of ρ in the corresponding BCPs and also by the values of the NCI isosurfaces located in these conventional hydrogen bonds formed by 4,7‐(OH)_2_phen and 4,7‐(NH_2_)_2_phen intercalators. The accuracy of the values for the interaction energies for such intercalation processes with 4,7‐(OH)_2_phen and 4,7‐(NH_2_)_2_phen intercalators has been also corroborated with benchmark calculations at DLPNO‐CCSD(T)/cc‐pVTZ level. The results indicate that the DFT‐D method for the EDA at B3LYP‐D3/TZP level faithfully reproduce the interaction energies between the DNA ring models and the considered phen derivatives.

The inclusion of solvent effects produces an important penalty, Δ*E*_Solv_. Nevertheless, when adding this penalty to the Δ*E*_int_ we still obtain important negative energies although the inclusion of the solvent effects may alter the final trends of the systems when comparing the final values of the Δ*E*_aq_ energies to those of the Δ*E*_int_ of the original EDA, which emphasizes the importance of taking into account solvent effects for this kind of processes. Thus, cytotoxic effects of this kind of ligands will depend not only on the intrinsic forces derived from the EDA but also on the solvent effects.

We also observed that the nature of the studied ligands 4,7‐(NH_2_)_2_phen and 4,7‐(OH)_2_phen as electron acceptors/electron donors may be altered with the orientation of the intercalation process, through the mg or via MG, which would break with the classical consideration of the intercalators as electron acceptors.^46^, ^48^, ^49^, ^52^, ^58^ Nevertheless, we have to keep in mind that such charge transfer/polarization is very small as observed in the values of the charge on the intercalator and in the Δ*E*_orb_ of the EDA.

Finally, the main conclusion that arises from the MD simulations is not only the important role of the sugar and phosphate backbone in order to describe properly the intercalation of 4,7‐(NH_2_)_2_phen and 4,7‐(OH)_2_phen ligands but also the inclusion of more stacked bps in order to stabilize the model. That is, whereas the hydrogen bonds formed between the —NH_2_ and —OH functional groups and heteroatoms of the bps change and disappear during the time of the simulation, conventional hydrogen bonds formed with the sugar and phosphate backbone may remain along the time, particularly those formed by the —OH functional group present in the intercalator. Nevertheless, limitations have to be highlighted with respect to the ring model when performing MD simulations in a box of explicit water molecules since distortion of the ring model is produced with time due to the lack of the stabilization that the rest of bps would produce if they were added to the ring model.

## 4 COMPUTATIONAL DETAILS

In order to build our models, first of all, we considered the 4e1u structure from the Protein Data Bank (PDB). Because we are interested only in the intercalation, for this 4e1u structure we cleaned and removed all the atoms with the exception of the dppz intercalator and the surrounding atoms forming the intercalation pocket. That is, the GC/CG bps, sugars and phosphates. Sodium atoms were also added in order to balance the negative charges of the two phosphate groups and the dppz ligand was transformed manually into 4,7‐(OH)_2_phen and the ring model^56^ (intercalator + bps + sugars + phosphates) for the intercalation of 4,7‐(OH)_2_phen via mg was obtained. The intercalation of 4,7‐(OH)_2_phen between GC/CG bps through the MG was achieved by rotating the 4,7‐(OH)_2_phen ligand in such a way that the N atoms became in the side of the MG, whereas —OH groups were in the side of the mg (see Figure 2 to locate the side corresponding to the mg and MG in the bps). From these models we obtained the counterparts for the intercalation between AT/TA bps by transforming manually the GC/CG bps into AT/TA bps. A similar process was carried out to obtain the working models for the 4,7‐(NH_2_)_2_phen intercalator.

Because classical intercalators are flat ligands made from fused rings, they are very restricted to motion and their number of conformers is small. However, when substitution is produced with some group having single bonds, the degrees of freedom of these molecules increase and different conformations can be achieved due to single‐bond rotamers. This is the case of the hydroxyl group in 4,7‐(OH)_2_phen for which we have the torsions associated to the C—C—O—H dihedral angle of each —OH functional group. Moreover, since the surrounding DNA pocket has several heteroatoms with electronic lone pairs to form hydrogen bonds with the hydroxyl groups of the 4,7‐(OH)_2_phen ligand some conformations will be more stable than others. Therefore, to carry out some conformational search associated to such rotamers and to find which hydrogen bonds with DNA are more stable, the following strategy was applied. First of all, a systematic conformational search by rotating 45° each single bond associated to the C—C—O—H torsions of each —OH functional group was carried out. That is, we fixed one torsion for the —OH group in position 4 and we rotated the other —OH group in position 7 in steps of 45°. Thus, for one fixed C—C—O—H torsion angle for the —OH in position 4 we obtained eight conformations by rotating the C—C—O—H torsion angle of the —OH in position 7. Once we had these eight conformations, we rotated 45° the torsion angle of the —OH (4) and we obtained the eight conformations corresponding to the —OH (7). This process was repeated until complete the eight conformations for the —OH (4) and it gave us a total of 64 conformations associated to the C—C—O—H rotations of the two —OH functional groups (see Figure S1). Because a similar situation is found for the C—C—N—H torsion angle of the amino group, the same process of systematic conformational search was applied for the 4,7‐(NH_2_)_2_phen ligand. Subsequently, all the structures obtained from the systematic conformational search were optimized at PM6‐DH2 level.^97^ We chose this semiempirical method because it takes into account corrections to dispersion forces and it was observed in the bibliography and in our previous works that it describes well this kind of systems in which dispersion forces are important.^30^, ^33^, ^35^, ^98^ Once we optimized all the conformations at PM6‐DH2 level we just took into account the most stable structure of each studied system forming several hydrogen bonds with DNA for our subsequent analyses. All semiempirical calculations were carried out with the MOPAC software.^99^ Net atomic charges were calculated by taking into account the Hirshfeld scheme,^100^ which was observed to perform correctly for this kind of systems.^32^ Localization of BCPs^60^ to perform QTAIM topologies and the NCI^65^ analyses were carried out by using the AIMALL software package.^101^ Hirshfeld charges and the wave function to perform the topological calculations were generated at M06‐2X/6–31 + G(d,p) level from the optimized PM6‐DH2 geometries with Gaussian software.^102^

The so‐called EDA^103^ for the intercalated systems was also carried out with the ADF software^104^, ^105^, ^106^ in order to calculate the contribution of the different kind of energies to the interaction energy between the considered fragments: intercalator and intercalation pocket (bps, sugars, phosphates and sodium atoms). The EDA splits the interaction energy into contributions related to orbital, Pauli, and electrostatic contributions following a Morokuma‐type energy decomposition method.^103^, ^107^ Because it is more useful for the discussion to have an explicit Δ*E*_disp_ term for dispersion the results of the EDA are reported with the B3LYP‐D3 functional with the explicit Grimme's D3 correction to dispersion contribution^108^, ^109^, ^110^, ^111^ and the TZP basis set. Solvent effects were described by the continuous solvent model COSMO^112^ as implemented in ADF software.^104^, ^105^, ^106^ The interaction energies obtained from the EDA were compared with benchmark DLPNO‐CCSD(T) calculations in which the cc‐pVTZ basis set was used and the RIJCOSX approximation for coulomb integrals and HF exchange along with the cc‐pVTZ/C and def2/J auxiliary basis sets. These benchmark DLPNO‐CCSD(T) calculations were performed with ORCA software.^113^, ^114^, ^115^

We also determined the stability of the conventional hydrogen bonds during the time by performing 2 ps semi‐empirical MD simulations with the PM6‐DH2 Hamiltonian. We chose this time of simulation because bond vibrations and even chemical reactions may be appreciated within this time of simulation and in the case of the conventional hydrogen bonds studied in this work this scale of simulation should be enough to monitories their stability during time.^94^, ^95^, ^96^ The time step of the MD was 1 fs and snapshots were taken from the simulation during each step. It must be said that for these simulations and because the implicit solvent approach does not take into account the formation of eventual competing hydrogen bonds with the solvent, explicit water molecules were considered. Such explicit water molecules were put considering the TIP3PBOX solvent model with Chimera 1.13^116^ tools by means of a rectangular box with edges no closer than 5 Å to any atom of the solute. To see the effect of the stacking of the rest of the bps in the ring model, we also carried out MD simulations with the 4,7‐(OH)_2_phen ligand intercalating via mg with the d(AATT)_2_ DNA tetramer obtained from the 4e1u PDB structure in a similar way that we obtained the intercalating ring systems before. The system was also put in a rectangular water box with edges no closer than 5 Å to any atom of the d(AATT)_2_ DNA tetramer intercalating the 4,7‐(OH)_2_phen ligand via mg. As for the ring models, the TIP3PBOX solvent model was considered with Chimera 1.13. Prior to simulation of 2 ps a relaxation pre‐process was carried out in which the d(AATT)_2_ DNA tetramer intercalating the 4,7‐(OH)_2_phen ligand was fixed along with the Na^+^ atoms and only H_2_O molecules were allowed to move in a pre‐simulation of 0.5 ps with time‐steps of 1 fs. Subsequently, and in order to minimize non‐desirable perturbations of the Na^+^ cations to the solute (DNA ring model + intercalator) that could affect the MD simulations, the Na^+^ cations were allowed to move along with H_2_O molecules during 0.5 ps with time‐steps of 1 fs and only the d(AATT)_2_ DNA tetramer intercalating the 4,7‐(OH)_2_phen ligand was fixed. After this pre‐process of relaxation the d(AATT)_2_ DNA tetramer intercalating the 4,7‐(OH)_2_phen ligand via mg was also allowed to move with Na^+^ atoms and H_2_O molecules to perform the MD simulation of 2 ps with time‐steps of 1 fs.

## Supporting information


**Data S1.** Supporting information.

## Data Availability

The data that supports the finding of this study are available in the Supporting information of this article.
