# Supplementary material for: Influence of conventional hydrogen bonds in the intercalation of phenanthroline derivatives with DNA: The important role of the sugar and phosphate backbone
Source: J Comput Chem. 2022 Mar 17;43(11):804–21. doi: 10.1002/jcc.26836 (PMC9313584; doi:10.1002/jcc.26836)
Supplement: Supplementary file 1 — Data S1. Supporting information. [file JCC-43-804-s001.docx]

**Supporting information**

**Influence of Conventional Hydrogen Bonds in the Intercalation of Phenanthroline Derivatives with DNA: The Important Role of the Sugar and Phosphate Backbone**

Ángel Sánchez-González^1^, Pierre Grenut^1^, Adrià Gil^1,2,3,*^

*^1^ BioISI – Biosystems and Integrative Sciences Institute, Departamento de Química e Bioquímica. Faculdade de Ciências, Universidade de Lisboa, Campo Grande, Lisbon, Portugal*

*^2^ ARAID Foundation, Zaragoza, Spain*

*^3^ Departamento de Química Inorgánica, Instituto de Síntesis Química y Catálisis Homogénea (ISQCH) CSIC-Universidad de Zaragoza, c/ Pedro Cerbuna 12, Zaragoza, Spain*

**Index**

**Figure S1.** Schematic representation of the systematic conformational search by changing the C-C-O-H dihedral angle by steps of 45º a similar procedure was used for the C-C-N-H dihedral angle. 3

**Figure S2.** Complete bonding scheme from QTAIM topological analysis of ρ for 4,7-(NH_2_)_2_phen, intercalated between AT/TA base pairs via Minor Groove. For the considered interactions the corresponding BCPs, ρ (a.u.) and E_d_ (a.u.) are presented. 4

**Figure S3.** Complete bonding scheme from QTAIM topological analysis of ρ for 4,7-(OH)_2_phen, intercalated between AT/TA base pairs via Minor Groove. For the considered interactions the corresponding BCPs, ρ (a.u.) and E_d_ (a.u.) are presented. 5

**Figure S4.** Complete bonding scheme from QTAIM topological analysis of ρ for 4,7-(NH_2_)_2_phen, intercalated between AT/TA base pairs via Major Groove. For the considered interactions the corresponding BCPs, ρ (a.u.) and E_d_ (a.u.) are presented. 6

**Figure S5.** Complete bonding scheme from QTAIM topological analysis of ρ for 4,7-(OH)_2_phen, intercalated between AT/TA base pairs via Major Groove. For the considered interactions the corresponding BCPs, ρ (a.u.) and E_d_ (a.u.) are presented. 7

**Figure S6.** Complete bonding scheme from QTAIM topological analysis of ρ for 4,7-(NH_2_)_2_phen, intercalated between GC/CG base pairs via Minor Groove. For the considered interactions the corresponding BCPs, ρ (a.u.) and E_d_ (a.u.) are presented. 8

**Figure S7.** Complete bonding scheme from QTAIM topological analysis of ρ for 4,7-(OH)_2_phen, intercalated between GC/CG base pairs via Minor Groove. For the considered interactions the corresponding BCPs, ρ (a.u.) and E_d_ (a.u.) are presented. 9

**Figure S8.** Complete bonding scheme from QTAIM topological analysis of ρ for 4,7-(NH_2_)_2_phen, intercalated between GC/CG base pairs via Major Groove. For the considered interactions the corresponding BCPs, ρ (a.u.) and E_d_ (a.u.) are presented. 10

**Figure S9.** Complete bonding scheme from QTAIM topological analysis of ρ for 4,7-(OH)_2_phen, intercalated between GC/CG base pairs via Major Groove. For the considered interactions the corresponding BCPs, ρ (a.u.) and E_d_ (a.u.) are presented. 11

**Movies** 12

**Cartesian coordinates for the optimized ring model systems at PM6-DH2 level** 14

| 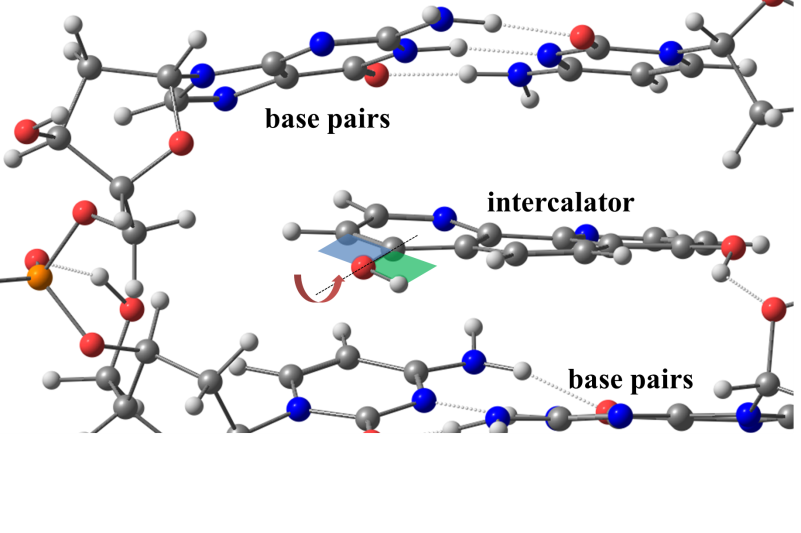 | 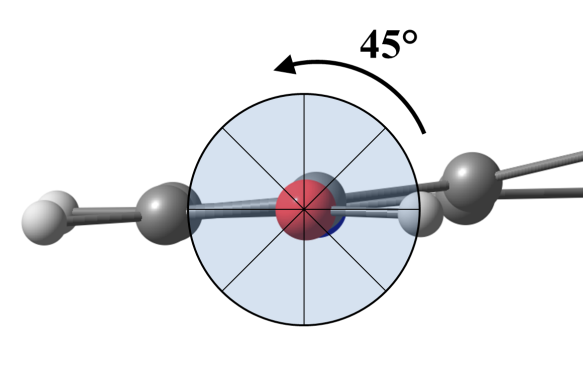 |
| --- | --- |

**Figure S1.** Schematic representation of the systematic conformational search by changing the C-C-O-H dihedral angle by steps of 45º a similar procedure was used for the C-C-N-H dihedral angle.

**Complete Bonding secheme for QTAIM topological analysis of electron density for the considered ring models**


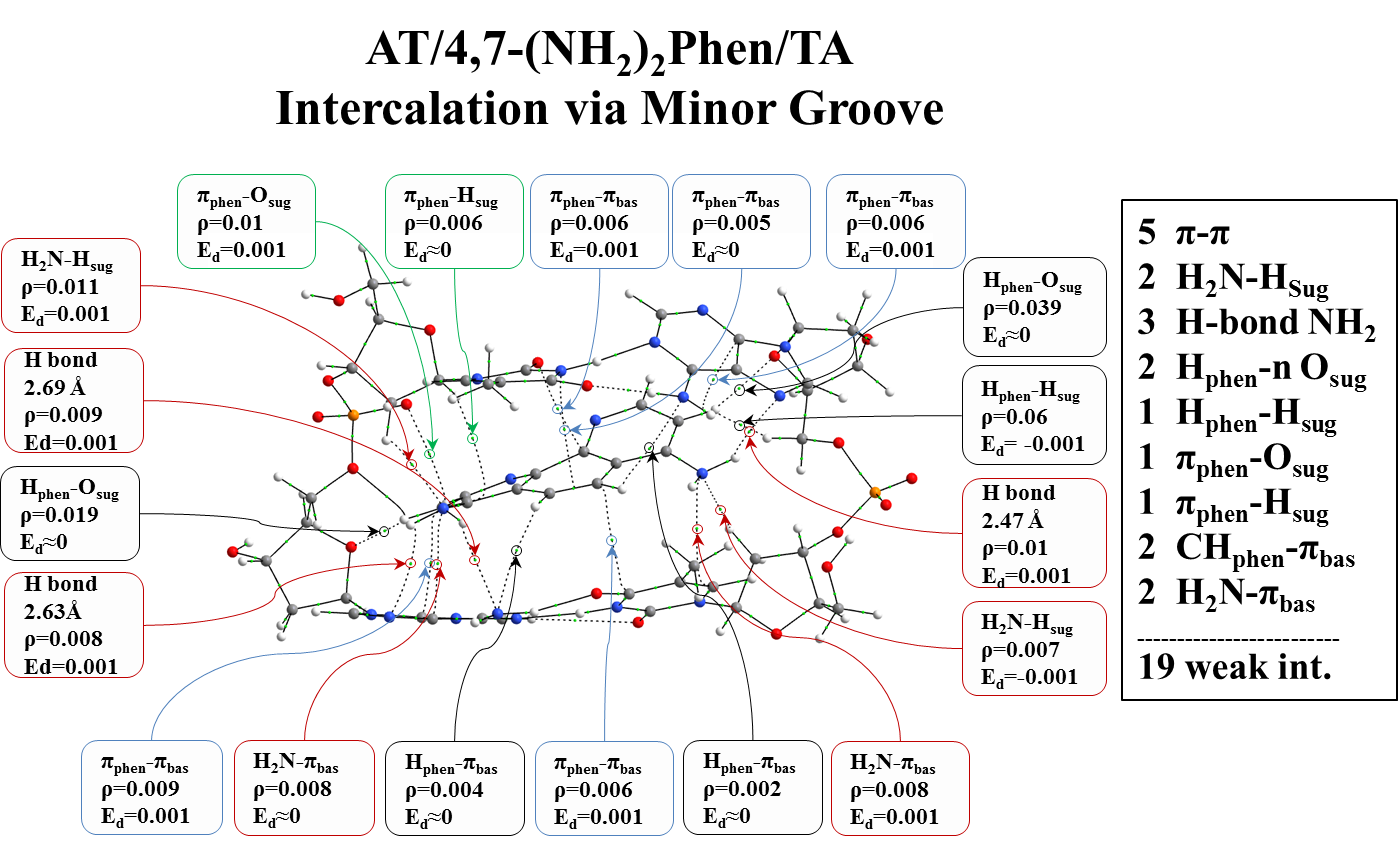


**Figure S2.** Complete bonding scheme from QTAIM topological analysis of ρ for 4,7-(NH_2_)_2_phen, intercalated between AT/TA base pairs via Minor Groove. For the considered interactions the corresponding BCPs, ρ (a.u.) and E_d_ (a.u.) are presented.


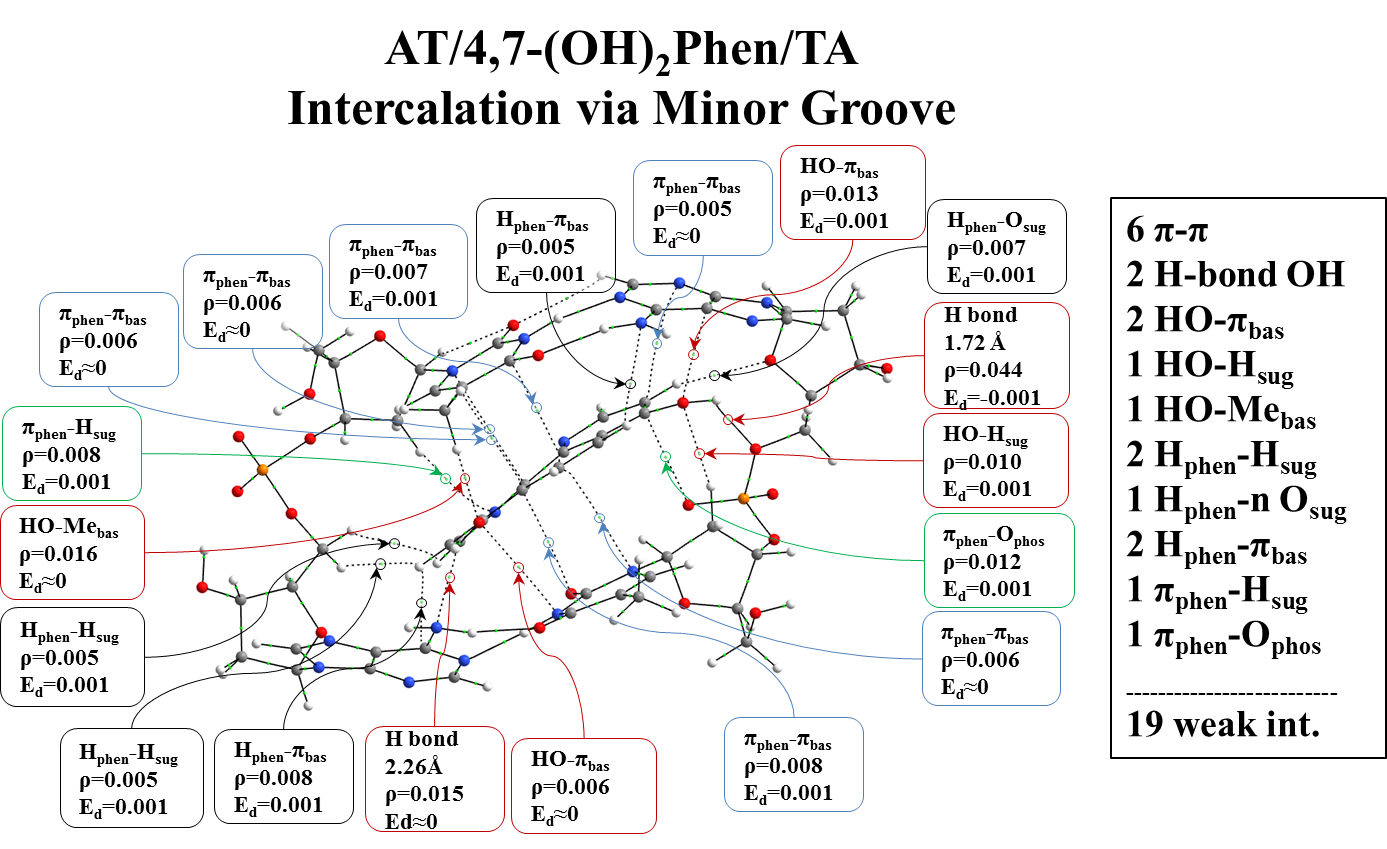


**Figure S3.** Complete bonding scheme from QTAIM topological analysis of ρ for 4,7-(OH)_2_phen, intercalated between AT/TA base pairs via Minor Groove. For the considered interactions the corresponding BCPs, ρ (a.u.) and E_d_ (a.u.) are presented.


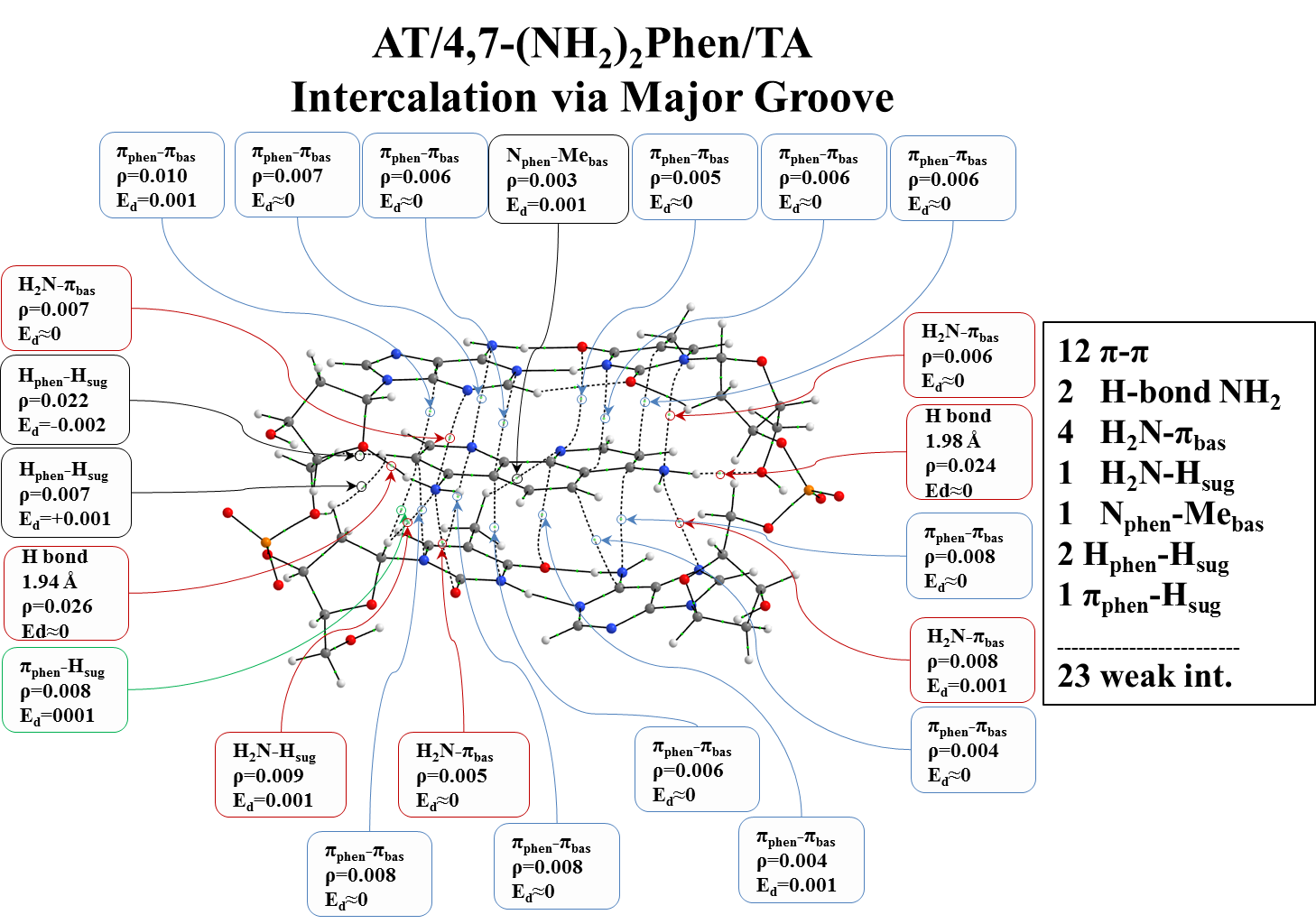


**Figure S4.** Complete bonding scheme from QTAIM topological analysis of ρ for 4,7-(NH_2_)_2_phen, intercalated between AT/TA base pairs via Major Groove. For the considered interactions the corresponding BCPs, ρ (a.u.) and E_d_ (a.u.) are presented.


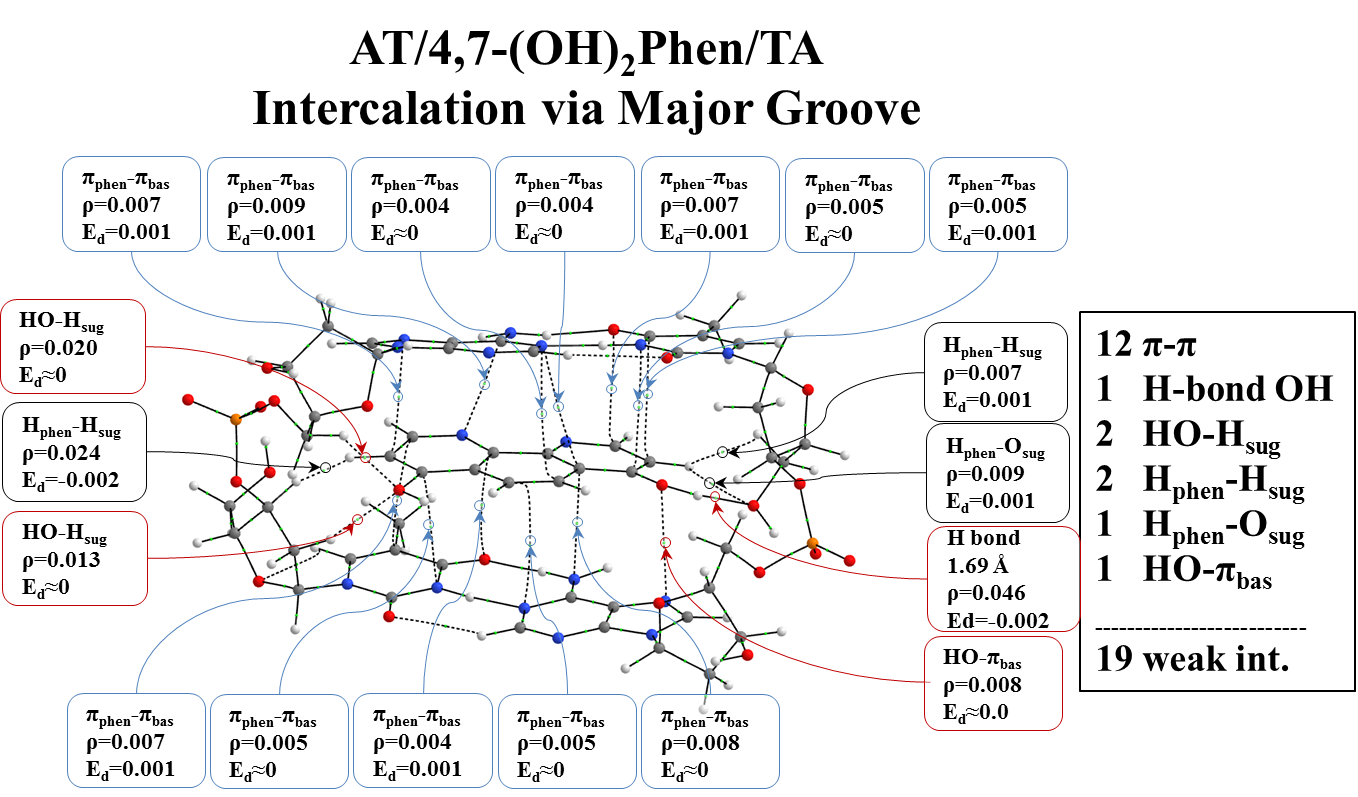


**Figure S5.** Complete bonding scheme from QTAIM topological analysis of ρ for 4,7-(OH)_2_phen, intercalated between AT/TA base pairs via Major Groove. For the considered interactions the corresponding BCPs, ρ (a.u.) and E_d_ (a.u.) are presented.


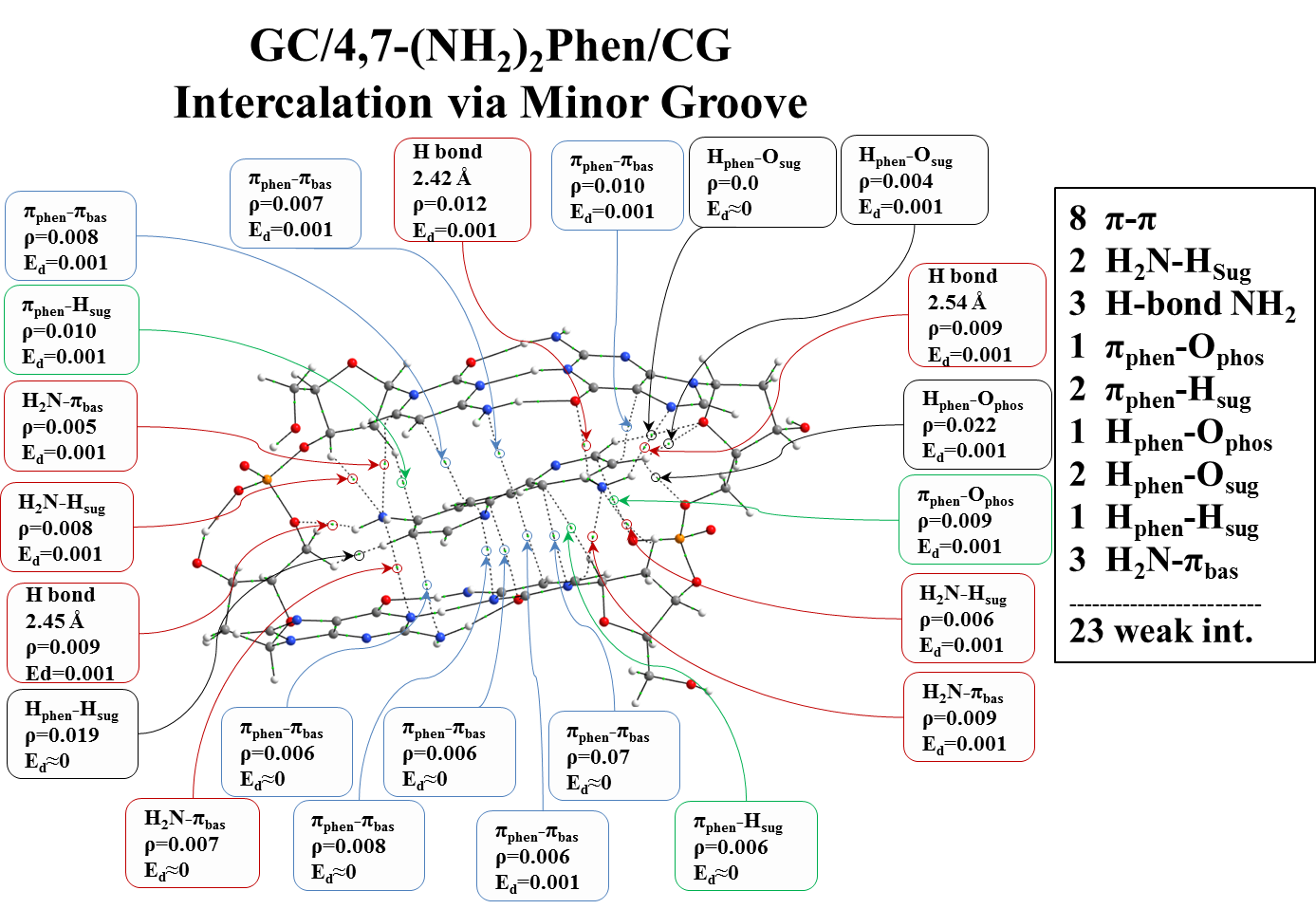


**Figure S6.** Complete bonding scheme from QTAIM topological analysis of ρ for 4,7-(NH_2_)_2_phen, intercalated between GC/CG base pairs via Minor Groove. For the considered interactions the corresponding BCPs, ρ (a.u.) and E_d_ (a.u.) are presented.


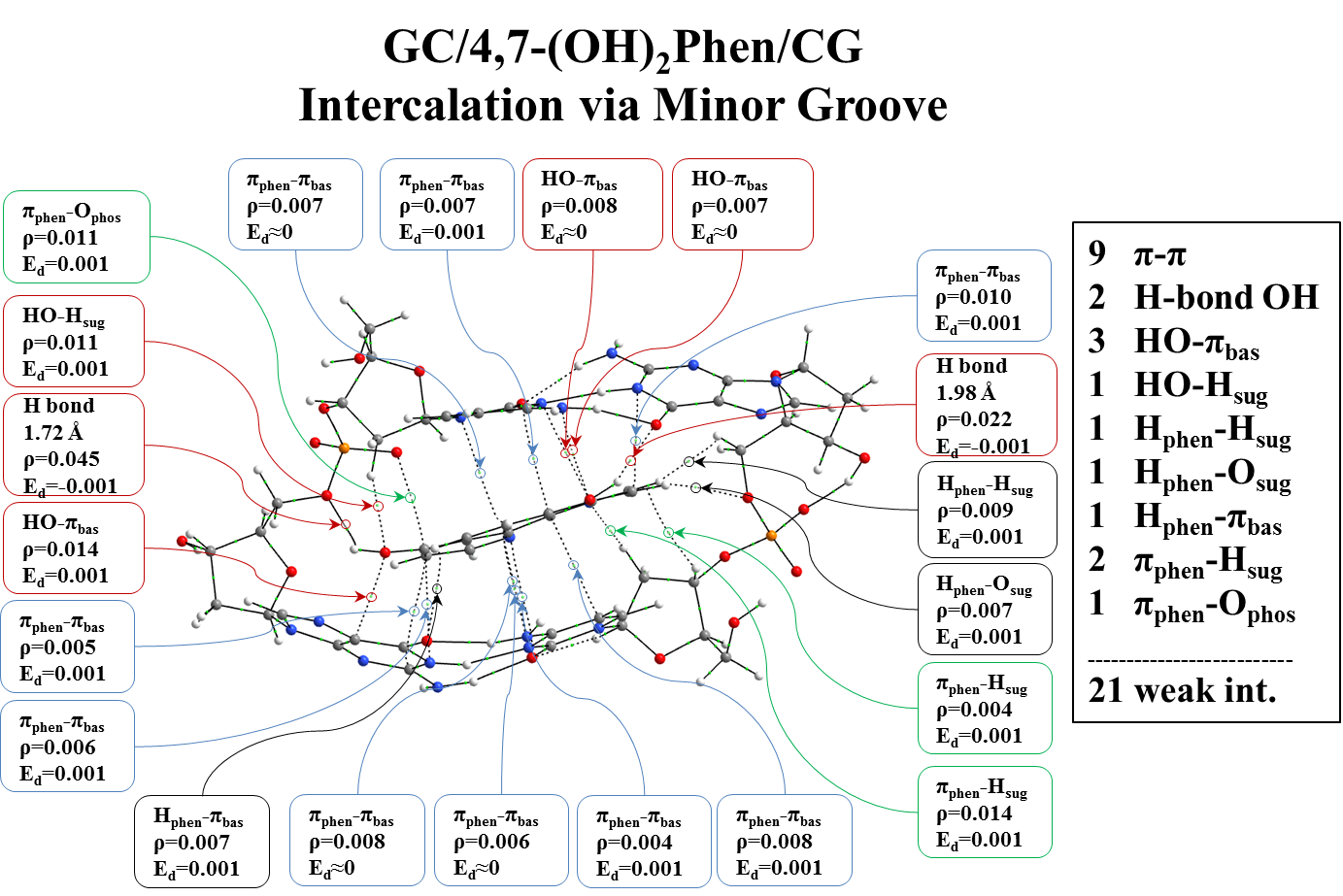


**Figure S7.** Complete bonding scheme from QTAIM topological analysis of ρ for 4,7-(OH)_2_phen, intercalated between GC/CG base pairs via Minor Groove. For the considered interactions the corresponding BCPs, ρ (a.u.) and E_d_ (a.u.) are presented.


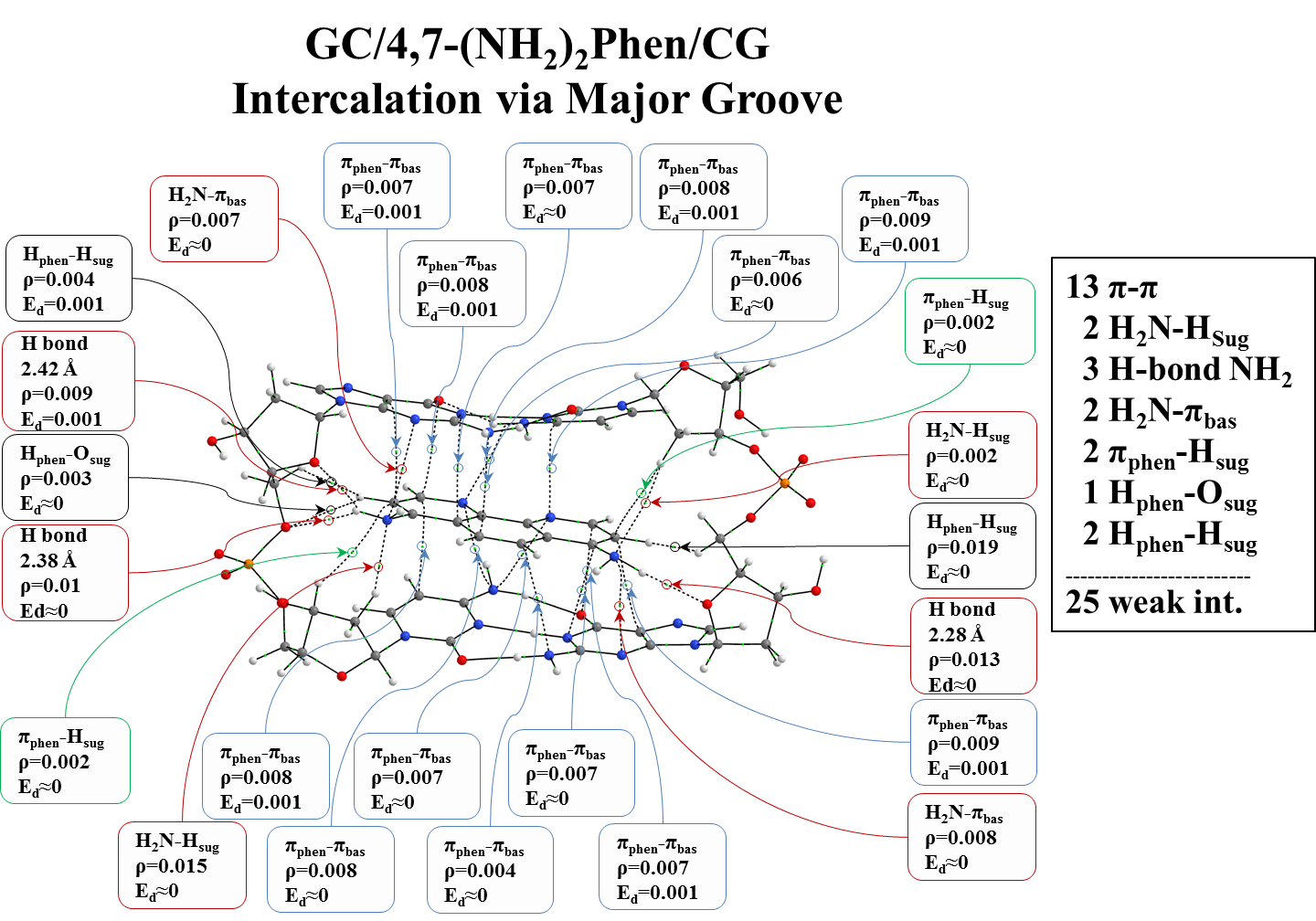


**Figure S8.** Complete bonding scheme from QTAIM topological analysis of ρ for 4,7-(NH_2_)_2_phen, intercalated between GC/CG base pairs via Major Groove. For the considered interactions the corresponding BCPs, ρ (a.u.) and E_d_ (a.u.) are presented.


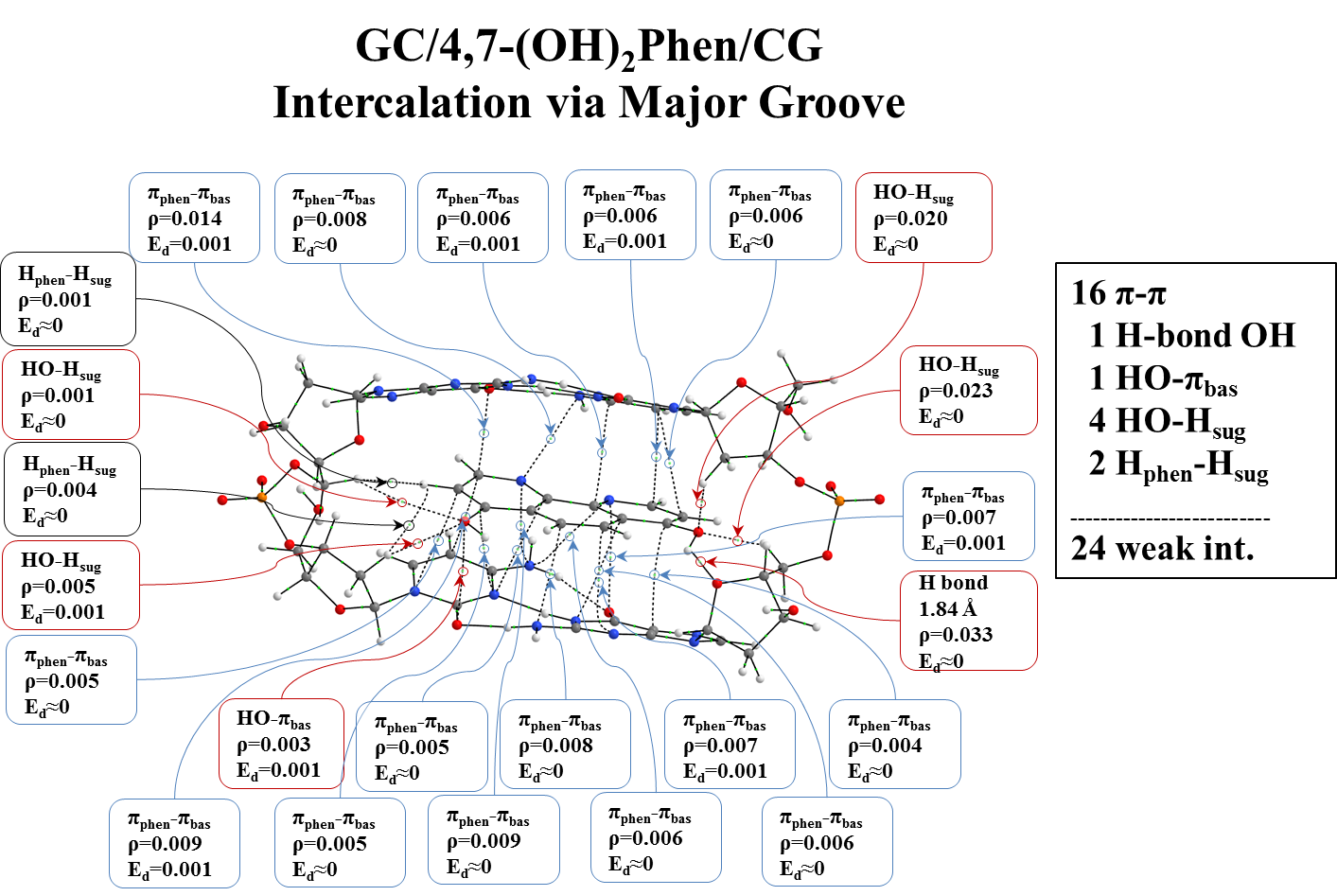


**Figure S9.** Complete bonding scheme from QTAIM topological analysis of ρ for 4,7-(OH)_2_phen, intercalated between GC/CG base pairs via Major Groove. For the considered interactions the corresponding BCPs, ρ (a.u.) and E_d_ (a.u.) are presented.

**Movies**

[**https://www.dropbox.com/s/mcuxwty3s806n3o/ATTAphen47NHmaj.mp4?dl=0**](https://www.dropbox.com/s/mcuxwty3s806n3o/ATTAphen47NHmaj.mp4?dl=0)

**(AT/4,7-(NH_2_)_2_phen/TA)mg**

[**https://www.dropbox.com/s/akjre70sniaepy5/attaphen47ohmin.mp4?dl=0**](https://www.dropbox.com/s/akjre70sniaepy5/attaphen47ohmin.mp4?dl=0)

**(AT/4,7-(OH)_2_phen/TA)mg**

[**https://www.dropbox.com/s/mcuxwty3s806n3o/ATTAphen47NHmaj.mp4?dl=0**](https://www.dropbox.com/s/mcuxwty3s806n3o/ATTAphen47NHmaj.mp4?dl=0)

**(AT/4,7-(NH_2_)_2_phen/TA)MG**

[**https://www.dropbox.com/s/a2w08aac36w8lse/ATTAphen47OHmaj.mp4?dl=0**](https://www.dropbox.com/s/a2w08aac36w8lse/ATTAphen47OHmaj.mp4?dl=0)

**(AT/4,7-(OH)_2_phen/TA)MG**

[**https://www.dropbox.com/s/yx5iw7c3o73zvt0/gccgphen47nhmin.mp4?dl=0**](https://www.dropbox.com/s/yx5iw7c3o73zvt0/gccgphen47nhmin.mp4?dl=0)

**(GC/4,7-(NH_2_)_2_phen/CG)mg**

[**https://www.dropbox.com/s/shgtv2updmezeak/gccgphen47ohmin.mp4?dl=0**](https://www.dropbox.com/s/shgtv2updmezeak/gccgphen47ohmin.mp4?dl=0)

**(GC/4,7-(OH)_2_phen/CG)mg**

[**https://www.dropbox.com/s/x7bk6npl1gurxdj/gccgphen47nhmaj.mp4?dl=0**](https://www.dropbox.com/s/x7bk6npl1gurxdj/gccgphen47nhmaj.mp4?dl=0)

**(GC/4,7-(NH_2_)_2_phen/CG)MG**

[**https://www.dropbox.com/s/sb2qchbhoobak46/gccgphen47ohmaj.mp4?dl=0**](https://www.dropbox.com/s/sb2qchbhoobak46/gccgphen47ohmaj.mp4?dl=0)

**(GC/4,7-(OH)_2_phen/CG)MG**

[**https://www.dropbox.com/s/x8wqo9phemuazdl/TETRAMERO-ATTA.mp4?dl=0**](https://www.dropbox.com/s/x8wqo9phemuazdl/TETRAMERO-ATTA.mp4?dl=0)

**d(AATT)2 tetramer intercalating 4,7-(OH)_2_phen/CG through AT/TA via mg**

**Cartesian coordinates for the optimized ring model systems at PM6-DH2**

**(AT/4,7-(NH_2_)_2_phen/TA)mg**

C 1.91582150 6.48218982 27.93137824

C 1.17557583 7.61480096 27.47377392

C 1.64152176 8.91241568 27.88550549

N 2.78386903 9.13608427 28.61495665

C 3.42653487 8.00045659 28.98257071

N 3.04855934 6.71236919 28.68573576

N -0.02500505 7.74353519 26.75154550

C -0.30256693 9.05006919 26.73772832

N 0.70114884 9.83919569 27.40027705

C 0.58940548 11.22950127 27.92547570

O -0.06157650 11.16367989 29.19730274

C -1.44152152 11.61400275 29.12748409

C -1.58562735 12.36459914 27.77985160

C -0.24809657 12.14709827 27.03298246

C -2.39989446 10.41572426 29.25069777

O -2.11315947 9.61492778 30.38854248

P -2.49396016 10.24420207 31.93332212

O -2.59253293 11.71723114 31.87203141

O -1.84260881 13.74748659 27.96481013

N 1.49682991 5.18958482 27.75643693

O -4.08401228 9.66543004 32.00394860

C -4.41774427 8.30483674 32.14719802

C -3.40348861 7.27032605 31.62445522

C -2.63946317 6.79529102 32.86080089

O -3.41343578 7.16873239 34.02761765

C -4.57897648 7.95161553 33.65336323

C -5.80052034 7.07331231 33.98313883

O -6.66215834 6.88208391 32.85485877

N -2.46999651 5.29942896 32.95103995

C -1.49213529 4.79182873 33.86718077

N -1.32650287 3.40074861 33.89811390

C -2.03041922 2.46719494 33.08001574

C -3.13929183 3.05127752 32.31741634

C -3.32757938 4.39871016 32.29047834

O -0.81900754 5.51397325 34.58986791

O -1.63602685 1.31166025 33.10867158

C -4.04063764 2.09914785 31.61101216

O -1.62792025 9.41143033 32.83456520

N -0.80330004 6.52559819 29.79525127

C 0.16371096 7.10561433 30.64913278

C 0.91230918 6.28816495 31.56023202

C 1.64255785 6.96652063 32.57440017

N 1.83471671 8.33331431 32.58799849

C 1.21965936 9.06278097 31.63007066

C 0.36006743 8.48763599 30.67283396

C 2.21847490 6.15520274 33.66029600

C 2.48503317 4.78771853 33.39776872

C 1.84449670 4.15213141 32.27463542

C 1.03333199 4.85547628 31.44130281

N 2.45118056 6.74194779 34.88430675

C 3.11336080 6.01137709 35.80065075

C 3.62679606 4.72106240 35.54614244

C 3.33982028 4.10107421 34.32598628

N 3.97421473 2.90539216 33.95786682

N 0.99551204 0.77340331 34.44565209

C 1.28636887 1.48228669 35.57734111

C 2.47097448 1.31906222 36.35451068

C 2.62678972 2.17163052 37.49900580

N 1.72195370 3.11689638 37.91028654

C 0.65950316 3.23933975 37.07456977

N 0.41249447 2.48597003 35.95623098

N 3.61311719 0.50735436 36.20294528

C 4.44631944 0.84991030 37.18334478

N 3.87645188 1.84776808 38.06035608

C 4.48708003 2.66519762 39.12580079

O 4.95941717 3.90047095 38.55102784

C 6.39688443 3.85809889 38.35565017

C 6.90530537 2.90368534 39.47001602

C 5.69889576 2.00318797 39.80795715

C 6.67533305 3.40770761 36.91343753

O 6.93465951 2.00604346 36.89276177

P 8.39285726 1.47236262 36.18234584

O 9.37651031 1.68209042 37.30679906

O 7.27439178 3.67719009 40.62747398

O 8.64471462 2.70637692 35.08404771

C 7.84575978 2.57361247 33.89424932

C 8.74620346 2.04001819 32.75227673

O 8.46787061 2.88067450 31.59010434

C 7.57337118 3.95071885 31.91978776

C 7.42647894 3.97857935 33.44763305

N 6.26118204 3.68027518 31.23860999

C 5.65171871 2.42008156 31.28534454

C 4.47142090 2.17145248 30.65136215

C 3.80670679 3.20766317 29.86180393

N 4.43211177 4.48605775 29.88843886

C 5.63618702 4.77859927 30.55328956

O 6.13457328 5.88900970 30.55885325

O 2.78342270 3.09422389 29.19470740

C 3.82307131 0.83120139 30.70315461

C 8.47450554 0.58141354 32.34743403

O 7.24919552 0.10108492 32.89884108

O 8.09373982 0.17805099 35.52026955

Na 9.37676812 2.24604706 39.32106019

Na -0.44074136 7.77569037 33.79215293

H 4.34821700 8.10102243 29.59068488

H -0.08619424 4.02337735 37.30376393

H 3.27207611 6.47741663 36.78275925

H 1.99452849 3.08161712 32.13146351

H -5.47134807 6.04210883 34.23198718

H -6.38014511 7.48129945 34.82799673

H -4.53216686 8.85559614 34.30224362

H -5.38712879 8.23603949 31.59353312

H -3.94670153 6.46070295 31.09394625

H -2.73965319 7.69610166 30.82901130

H -1.60075765 7.28255454 33.03606744

H -4.15903292 4.85614929 31.72577924

H -3.44972008 10.77177524 29.27481777

H -2.26952611 9.70304514 28.41285723

H -1.53739933 12.28995665 30.02335490

H -2.46053222 12.04196550 27.17554808

H -0.40589468 11.75877184 26.01326592

H 0.25850643 13.12220808 26.88116624

H 1.62526083 11.58633522 28.16280646

H -1.15455114 9.51117442 26.25814624

H 8.33075238 0.50506627 31.24994494

H 9.30550263 -0.07612934 32.66272937

H 9.82446126 2.21523540 32.96864313

H 6.94842015 1.92280798 34.05900934

H 6.39970686 4.23661050 33.76969638

H 8.08830940 4.74417336 33.89934107

H 8.02193567 4.86982637 31.46468455

H 6.17855095 1.63505038 31.86320237

H 5.73079661 3.54456547 36.31895265

H 7.46921539 4.01595111 36.42873419

H 6.68040465 4.92842305 38.51104070

H 7.88877968 2.36426359 39.22562558

H 5.84088804 0.95508962 39.47585402

H 5.53217577 1.93893196 40.90457562

H 3.67522082 3.00173145 39.82364728

H 5.44645972 0.45236344 37.33670344

H 4.23118415 4.22635952 36.30973185

H 4.34462958 2.31695078 34.69627097

H 0.50553453 4.35287407 30.62776075

H -0.56872314 5.60908723 29.42405641

H -0.15817539 9.15912886 29.96252376

H 1.44305219 10.14621152 31.60326169

H -1.27601235 14.13224373 28.67073620

H 7.38302151 -0.07867170 33.89788980

H 6.53338646 4.26305273 40.91797098

H -7.22937796 7.67018814 32.72207642

H -4.57815362 2.56170394 30.77427719

H -3.47648025 1.24256874 31.20438607

H -4.79223020 1.68248561 32.29994708

H -0.64767970 3.02708352 34.63315713

H 1.56467643 -0.02228566 34.19304714

H 0.07501761 0.87815771 33.98272675

H 4.15862282 0.23248470 31.56096750

H 2.72699148 0.91675606 30.74473227

H 4.06038435 0.25223703 29.79565085

H 3.95396572 5.30702282 29.39611199

H 0.83825183 4.97611719 27.02178392

H 2.05342019 4.40263363 28.14422230

H 3.54574894 2.36307721 33.21496538

H -1.13527287 7.14274823 29.05424547

**(AT/4,7-(OH)_2_phen/TA)mg**

C 5.01290305 3.31451133 31.59796960

N 5.45556540 4.64837569 31.48921321

C 5.00305243 5.47645066 30.40664387

N 4.09048803 4.88417549 29.51747978

C 3.55339382 3.57243257 29.63458437

C 4.09780075 2.78197822 30.74565548

C 6.57466607 5.18803147 32.32383458

O 7.78735677 4.52345697 31.93453975

C 8.16375124 3.49887566 32.89613353

C 7.18853922 3.64535222 34.09589147

C 6.38094145 4.92130069 33.82027653

O 7.93040547 3.80701339 35.30852714

P 8.09108965 2.34647378 36.18712288

O 8.67705372 1.30491666 35.30385070

C 8.08504258 2.13157480 32.18329701

O 7.30740573 1.19679555 32.92212853

O 5.38095514 6.62825372 30.28375861

O 2.70985385 3.23957182 28.81151226

C 3.58824354 1.39265045 30.90622904

O 6.52295470 1.88851219 36.45719528

C 5.63123598 2.75535633 37.16615022

C 5.96927183 2.83293390 38.66212460

O 4.71132335 3.08790022 39.34844258

C 4.31239933 1.93542740 40.10014125

C 5.60246325 1.17767213 40.42903708

C 6.54945533 1.51753537 39.26044656

N 3.39113820 1.14094417 39.23030422

C 2.26689323 1.68221290 38.58813154

C 1.87205030 0.71684867 37.59280117

N 2.73852709 -0.38950879 37.62626285

C 3.64132363 -0.11551447 38.56828068

N 1.61748728 2.87470743 38.80202898

C 0.58834909 3.09855958 37.95439845

N 0.14973141 2.26165732 36.95063706

C 0.76907728 1.04795115 36.75651976

N 0.38361206 0.28087562 35.67057575

O 7.84612868 1.74775912 39.79594189

O 8.73826266 2.91049322 37.44892435

O 1.50881011 2.08595724 33.29769306

C 2.00996419 3.25838692 33.77499806

C 1.88834612 4.38399268 32.89754444

C 2.46374958 5.60000584 33.34307828

N 2.96253421 5.75720508 34.62448724

C 3.00523808 4.68389360 35.42790132

C 2.59646849 3.38890785 35.02538543

C 1.17478954 4.31102035 31.64834256

C 0.99596023 5.42681968 30.89486164

C 1.57672004 6.68129469 31.29942721

C 2.42216794 6.76939377 32.44220291

N 3.09146237 7.92418571 32.78732619

C 2.90663940 9.02317678 32.02135499

C 2.01529542 9.07198538 30.93273438

C 1.32596193 7.90617130 30.60008100

O 0.41210125 7.84112224 29.60782588

C -2.42091795 7.01861179 31.57906214

C -3.46391699 8.08999512 31.96718942

C -3.84783189 7.70392118 33.42469789

O -2.65489463 7.09219973 33.97566239

C -1.82452732 6.56097902 32.91576072

O -3.07297454 9.44589919 31.99177607

P -1.47191105 9.96243771 31.84490734

O -0.53993102 9.00804389 32.53530081

N -1.81771065 5.06981340 33.13870913

C -1.09714658 4.59418621 34.28574299

N -1.25450988 3.24063679 34.61607801

C -2.03197421 2.30293705 33.86578692

C -2.84179542 2.89615795 32.79788638

C -2.74655268 4.21952265 32.50523613

C -5.00386807 6.69726507 33.53632570

O -5.20663850 5.96447666 32.31609803

O -0.35573770 5.31935717 34.93703407

O -1.95693473 1.13025985 34.19839343

C -3.76941173 1.99078330 32.06396060

O -1.35874115 9.47254575 30.19719543

C -2.02072857 10.31735566 29.25649022

C -1.14468215 11.54050748 28.94939496

O 0.19625139 11.08760019 28.63273616

C 0.46847543 11.19232971 27.22347756

C -0.59361282 12.10970260 26.61610678

C -1.64006677 12.35268540 27.72747615

N 0.45218221 9.81383713 26.67556802

C 1.46444814 8.89342201 27.01115093

C 0.91550397 7.58652786 26.77949291

N -0.40404668 7.69898720 26.30575787

C -0.67535722 9.00473705 26.27922964

N 2.72512201 9.15485600 27.48901941

C 3.41774613 8.03476822 27.81283957

N 2.97373020 6.74255928 27.70116942

C 1.71664890 6.47323281 27.18460571

N 1.31906923 5.17123515 27.11695839

O -1.75374632 13.73613027 28.02486217

O -1.48209613 11.43204592 31.94947751

Na 10.06630884 4.76656571 36.79498017

Na 0.43457958 7.40988517 33.76323942

H 4.43319483 8.15816270 28.23979295

H 0.03768404 4.05217615 38.05690886

H 3.38566717 4.84550133 36.44861643

H 0.79123922 3.33211575 31.34136035

H -4.74503330 5.90039031 34.26692513

H -5.94561191 7.18270798 33.84016459

H -4.01051327 8.59852661 34.06924875

H -4.33519681 8.08820783 31.27362515

H -2.93830044 6.20245718 31.02616215

H -1.65991812 7.38853050 30.84545670

H -0.76603329 6.96090396 33.14687153

H -3.39341379 4.69564734 31.74215638

H -3.01850224 10.62699686 29.63111742

H -2.14865908 9.65761463 28.37585545

H -0.99760827 12.17128165 29.86838882

H -2.67334957 12.09778521 27.40570047

H -1.05275186 11.70313523 25.69895034

H -0.14978455 13.07500735 26.29745187

H 1.52277537 11.57144690 27.17660291

H -1.60212808 9.45454154 25.95607274

H 7.55812271 2.22357758 31.21156444

H 9.09420629 1.71791965 32.01001788

H 9.21075422 3.75907796 33.15252328

H 6.49431824 2.75941128 34.16366906

H 5.31625971 4.81836304 34.10142405

H 6.74004417 5.77951814 34.42017786

H 6.73773583 6.26261411 32.04522085

H 5.44349097 2.71719433 32.41959865

H 4.65072560 2.25229437 37.00921557

H 5.58505543 3.75566483 36.69742920

H 6.59254896 3.71356142 38.92995099

H 6.60973031 0.69635888 38.51107271

H 5.47910319 0.09445953 40.57923356

H 6.04722332 1.55405107 41.37748988

H 3.72851295 2.34806280 40.95695157

H 4.46979669 -0.75162813 38.85500620

H 2.72343602 2.54107177 35.69061072

H 1.45845886 1.37425709 34.00725310

H 0.44518161 5.40488103 29.94679501

H 0.00922402 8.78637986 29.40436171

H 1.85830295 9.99756554 30.36777240

H 3.50790345 9.90751344 32.29081849

H -0.96753183 14.06990597 28.51235906

H 7.80511969 0.94908776 33.78442440

H 8.44684265 2.08692892 39.04220027

H -5.78418216 6.46784692 31.70780936

H -4.77426936 2.42466490 31.96438611

H -3.39450082 1.76676424 31.05431607

H -3.88291623 1.02293133 32.58142927

H -0.76648884 2.90251743 35.50373623

H 0.65488603 -0.69889052 35.67455417

H -0.53918323 0.47951746 35.23371740

H 4.35632693 0.70816812 31.29260685

H 2.73215846 1.36729909 31.60308925

H 3.23171423 0.98166987 29.94836727

H 3.74724751 5.50726374 28.71765476

H 0.39362228 4.94196759 26.78809729

H 1.87836752 4.42133422 27.56030021

**(AT/4,7-(NH_2_)_2_phen/TA)MG**

C 1.26170403 0.90232461 30.33094018

C 2.36951744 0.73091144 31.18735463

C 2.79649525 1.81470439 31.96231543

C 2.05978836 3.05340419 31.87616646

C 1.00479946 3.14338003 30.93196139

N 0.59144632 2.05906778 30.19057055

C 2.36512010 4.18156329 32.71433261

C 1.70142220 5.35781324 32.57818184

C 0.70653740 5.53617260 31.55309958

C 0.34074116 4.44393717 30.72795812

N -0.58477912 4.57224618 29.71155071

C -1.13132255 5.77871111 29.49211086

C -0.82291475 6.92705022 30.24725440

C 0.09685433 6.81634714 31.29620922

N 3.96463505 1.73818602 32.72249600

N 0.35221943 7.91796847 32.11842731

N 0.78228810 1.26268028 34.41664803

C 1.24472448 2.15883554 35.32189761

C 2.50388511 2.02612409 35.98419587

C 2.81521859 2.96625029 37.01983538

N 2.01040217 4.01536570 37.38859368

C 0.86380943 4.09564468 36.65725226

N 0.45356263 3.25162786 35.66290477

N 3.54125606 1.08279883 35.85421321

C 4.45267972 1.40901740 36.76469636

N 4.05435786 2.55669433 37.55214797

C 4.84150698 3.37321826 38.49130081

O 5.60355962 4.34851544 37.74906375

C 6.98121961 3.92333393 37.60337401

C 7.25478940 3.04214341 38.85719409

C 5.86281204 2.55981084 39.31355692

C 7.11981917 3.17746997 36.26238668

O 7.21272215 1.78635210 36.55950061

P 8.36796079 0.80112233 35.76504892

O 7.64732068 -0.47130845 35.49243509

O 7.88268326 3.83260671 39.88257938

O 8.64289914 1.68290136 34.38294615

C 7.62255264 1.55738815 33.36924227

C 7.87058304 0.29471212 32.50462291

O 8.11473202 0.76860542 31.14530612

C 7.81069242 2.16383157 31.02124870

C 7.84490496 2.75052102 32.43361500

N 6.46727588 2.25351520 30.33566527

C 5.67741450 3.44397071 30.41218250

N 4.53747695 3.48000590 29.59312815

C 4.08132617 2.41018071 28.77027437

C 4.87400358 1.17839845 28.86143200

C 6.01586814 1.14387047 29.59533801

O 3.10863123 2.61097928 28.05925861

O 5.99816985 4.39301723 31.11217100

C 4.37033565 -0.01384008 28.12473853

C 6.68190726 -0.68591402 32.45408597

O 5.75620559 -0.46857713 33.51527000

O 9.56379505 0.97243423 36.66613099

N 1.88413073 5.16557753 27.64287141

C 2.22367524 6.20946525 28.43954194

C 1.62235489 7.50781527 28.36203532

C 2.06983007 8.49505558 29.30291992

N 3.05646262 8.30509028 30.24055319

C 3.59738664 7.05636012 30.20235086

N 3.23035843 6.02821217 29.38212444

N 0.59319442 8.03466959 27.56380136

C 0.39430709 9.28150172 27.99301765

N 1.29482771 9.64106798 29.06011873

C 1.31922651 10.85180074 29.90282363

O 0.42731094 10.64051121 31.01727344

C -0.81067392 11.39003687 30.85937490

C -0.46562084 12.54243518 29.87876532

C 0.83704786 12.10789486 29.17107484

C -1.93051599 10.44736258 30.38962628

O -2.68937063 10.03112781 31.52154387

P -4.17035218 10.85936113 31.78423000

O -4.13687184 10.94969680 33.29407770

O -0.26873387 13.76208787 30.57999532

O -5.28223242 9.63592520 31.42649635

C -5.24951117 8.30770002 31.86663297

C -4.08498610 7.49674683 31.24845091

C -3.17037618 7.10349313 32.40283388

O -3.92916233 7.30165927 33.63531283

C -5.12560165 8.11361309 33.40923536

N -2.81133900 5.66672473 32.51726800

C -3.27775049 4.68463239 31.62071356

C -2.90179673 3.38784350 31.72654472

C -1.99141370 2.95807776 32.79934116

N -1.58371838 3.97917392 33.71210716

C -1.94499956 5.33469473 33.61643532

O -1.57933708 6.18985252 34.40423213

O -1.58127395 1.82472300 32.99247197

C -3.28474695 2.35897212 30.72446889

C -6.26146559 7.26506718 33.99851199

O -6.25485512 5.96549576 33.39316830

O -4.36780848 11.90573816 30.76887355

Na 9.49404407 1.78615124 38.68638025

Na -3.26235419 9.35876299 34.59216836

H 0.17961712 4.93405501 36.88549722

H 4.41273203 6.83491051 30.91865542

H 3.14392499 4.07232304 33.47032597

H -6.16583051 7.13233147 35.09435621

H -7.26845376 7.66254422 33.75776139

H -4.87346921 9.07116928 33.99480735

H -6.23779988 7.92626501 31.48631645

H -4.48177860 6.62578472 30.68393750

H -3.54898861 8.09749035 30.48015186

H -2.22940373 7.72509330 32.48828826

H -3.94200222 5.04041598 30.81835879

H -2.57355592 10.93029042 29.61919540

H -1.51188403 9.49532563 29.98222066

H -1.00871891 11.77239824 31.88755763

H -1.29267165 12.80061198 29.18020712

H 0.68170768 11.95705473 28.08800602

H 1.59463447 12.91655444 29.22288005

H 2.32727931 10.93329019 30.38708563

H -0.32564744 9.98505524 27.60114685

H 6.07930462 -0.54377353 31.53633610

H 7.03739649 -1.73242408 32.50113613

H 8.82441801 -0.21590646 32.76489217

H 6.60430070 1.56467379 33.82666474

H 7.10548991 3.56531994 32.59763415

H 8.83623937 3.21135055 32.63685038

H 8.58335203 2.56264546 30.32147422

H 6.65404183 0.24585755 29.64426152

H 6.17832333 3.31169490 35.67444565

H 7.96045680 3.55629089 35.64550175

H 7.52811832 4.89625772 37.60073509

H 8.04574564 2.22352572 38.69375401

H 5.71972684 1.46816390 39.17302677

H 5.70309395 2.73058181 40.39848099

H 4.13488473 4.00209904 39.09522143

H 5.39389265 0.89096602 36.93975206

H 0.39202604 13.66269523 31.29938795

H 6.24628069 -0.58155291 34.40923625

H 7.32793981 4.60433622 40.14615258

H -5.33473852 5.59774950 33.43522883

H -1.84267599 5.84215093 28.65836455

H -1.26429790 7.89080431 29.98951267

H 1.20094370 7.88863950 32.67315998

H 1.91828231 6.19204900 33.24491583

H 0.90138386 0.06312052 29.72179514

H 2.88657408 -0.22215075 31.22217059

H 4.06781703 2.39812236 33.48361778

H -0.11960526 1.40794189 33.92868580

H 1.37477121 0.50646425 34.09777744

H -0.91810512 3.71666549 34.50412788

H 2.25781062 4.21352142 27.82630562

H 1.05671054 5.22313139 27.06232845

H 3.98436239 4.39683171 29.59597009

H 4.08090346 -0.81727314 28.81640650

H 3.47258745 0.23370898 27.53026635

H 5.11859768 -0.41285993 27.42749933

H -4.23459252 2.57537600 30.22431101

H -2.49308620 2.29202218 29.94742272

H -3.35848391 1.35751829 31.17677213

H 0.18946308 8.84772436 31.71608581

H 4.36008386 0.81911660 32.95767588

**(AT/4,7-(OH)_2_phen/TA)MG**

C -1.66446265 5.19142544 30.29968201

C -1.62276769 6.27074585 31.21562249

C -0.53132801 6.34469361 32.06332560

C 0.50525150 5.36246710 32.03488409

C 0.32099143 4.27334205 31.14314515

N -0.73924844 4.22031742 30.26257516

C 1.30437829 3.17786968 31.16677926

C 2.43866287 3.30042213 32.01093870

C 2.63405899 4.47791785 32.81479959

C 1.69617164 5.45949579 32.83877426

N 1.07828238 2.05559282 30.39957570

C 3.07310169 1.03250164 31.33485665

C 3.33716845 2.18489307 32.06670295

C 1.93559422 1.02748186 30.49547878

O -0.51256333 7.41147075 32.92870147

O 4.43082308 2.31691722 32.85444168

N 0.71692339 1.63700330 34.19489807

C 1.22130712 2.47017460 35.13930671

C 2.48294541 2.27655921 35.78248919

C 2.86614140 3.22213282 36.78871991

N 2.11285185 4.29891498 37.18860278

C 0.92846948 4.39603947 36.52560833

N 0.46421048 3.56241781 35.54725710

N 4.10667031 2.77541402 37.28606146

C 4.42974409 1.59548711 36.51101054

N 3.47352310 1.28662936 35.64198486

H 0.25655655 5.23386365 36.79660052

C 4.94543698 3.57079294 38.19335831

O 5.75488719 4.48924666 37.42786474

C 7.10093787 3.98336515 37.26541897

C 7.33852605 3.07236669 38.50607902

C 5.93006360 2.71906254 39.02325618

C 7.19130426 3.25556026 35.91056622

O 7.17383274 1.85477001 36.18689637

P 8.20983503 0.79046466 35.35094197

O 7.36742576 -0.39281373 35.01731076

O 8.08693915 3.79030268 39.50539429

O 8.60743162 1.68513930 34.00701184

C 7.67534117 1.63018663 32.91142549

C 7.78459656 0.28592150 32.14311759

O 8.26290700 0.61525312 30.80546001

C 8.15968335 2.02670193 30.55850970

C 8.16117030 2.70307599 31.92978911

C 6.44131239 -0.45278541 31.99706556

O 5.54765984 -0.16729203 33.07555253

N 6.88773163 2.21499973 29.76723062

C 6.10967006 3.41463839 29.85951511

N 4.86815693 3.39208103 29.20825018

C 4.34528741 2.30503909 28.45295834

C 5.22793127 1.13250487 28.36802243

C 6.42936039 1.12352398 28.99918251

O 6.51467701 4.40691389 30.44415831

O 3.24684981 2.44208884 27.94003452

O 9.41328735 0.78089718 36.25729917

N 1.59521030 4.81490647 28.21395869

C 2.07617929 5.86266134 28.93312000

C 1.37761747 7.09538992 29.12939631

C 2.06058763 8.13964246 29.83534174

N 3.33064018 8.04602623 30.35196460

C 3.90624061 6.83389389 30.13295118

N 3.35615764 5.77349517 29.46797759

N 1.18360266 9.23971323 29.84434689

C -0.02368272 8.77836880 29.19065708

N 0.09521348 7.53031816 28.74852927

H 4.92745224 6.67184773 30.52997243

C 1.47799276 10.50564394 30.53228493

O 0.97458676 10.45829202 31.88094073

C -0.29803976 11.14181815 31.97861501

C -0.27823713 12.20709861 30.84102618

C 0.81588276 11.73092774 29.86606976

C -1.41776611 10.09336455 31.84597838

O -2.16705146 10.45343398 30.68857626

P -3.83215182 10.03448376 30.55806611

O -4.33902558 11.41130224 30.22905814

O 0.04192808 13.50000676 31.38113972

O -3.93865901 8.78125586 29.76521484

O -4.21335468 9.71363024 32.15921917

C -4.29291146 8.31345740 32.45404984

C -3.69682296 8.06661354 33.84411647

C -4.54036864 6.89412651 34.36838820

O -5.81712019 6.96724376 33.72352664

C -5.78145047 7.91528437 32.60457651

N -3.90228235 5.57676523 34.00341558

C -2.69481657 5.24716533 34.69789461

N -2.03595269 4.08631602 34.27222035

C -2.46340088 3.24453817 33.19827573

C -3.67141175 3.69641610 32.50370813

C -4.32955020 4.81906973 32.89739538

C -6.41994175 7.23028005 31.38778317

O -5.46270578 6.62324514 30.52963548

O -2.27877766 5.94765290 35.60925778

O -1.78866884 2.25225099 32.97010680

Na 9.41982219 1.58049828 38.28924566

Na -2.68917443 13.06390000 30.35416922

H 3.55032911 4.53339383 33.40756693

H -7.06203657 6.38583421 31.71573383

H -7.02651433 7.95338048 30.81256091

H -6.39728834 8.77526991 32.95116980

H -3.77010672 7.67407483 31.67955095

H -2.61002280 7.83941330 33.81277202

H -3.79316773 8.95985330 34.48869455

H -4.75019979 6.89112871 35.46276433

C -4.11716108 2.88033428 31.34052070

H -5.22794096 5.19034035 32.37512223

H -0.97373944 9.07620827 31.68052446

H -2.04263182 10.04236928 32.76346970

H -0.25181252 11.58282506 33.00230475

H -1.31176453 12.41172314 30.37551701

H 0.42975814 11.48492910 28.85655338

H 1.57375003 12.52300737 29.69132250

H 2.58821704 10.58843272 30.67806760

H -0.90357179 9.40735041 29.10487100

H 5.88545494 -0.12857820 31.09609166

H 6.59645253 -1.54736715 31.95773008

H 8.58888949 -0.37589309 32.53636045

H 6.63465938 1.83503896 33.27223325

H 7.54448580 3.62854914 31.98199277

H 9.18629997 3.03076871 32.20654498

H 9.02707593 2.26864350 29.90251614

C 4.73970023 -0.02530953 27.56895349

H 7.11293542 0.26079751 28.94735042

H 6.26860378 3.47339466 35.31444345

H 8.06937958 3.57407838 35.31172843

H 7.70496274 4.92235693 37.27259067

H 8.03784358 2.18098152 38.30995069

H 5.69295820 1.63789776 38.94177662

H 5.82116582 2.95385951 40.10233037

H 4.28409674 4.25311262 38.79167525

H 5.35319334 1.04580080 36.66523622

H 0.91694801 13.50486471 31.83634970

H 6.02155973 -0.36866393 33.97368259

H 7.62940736 4.61409147 39.79268880

H -4.98068818 7.34424052 29.99039985

H -2.48875949 5.12106272 29.57439547

H -2.42079473 7.01680690 31.21815875

H 0.29379151 7.39665210 33.51759896

H 1.82714379 6.34815465 33.45628067

H 1.71233638 0.15168014 29.87192068

H 3.71383404 0.15788432 31.41179083

H 4.89667934 1.39250399 33.03349314

H -0.18876668 1.83534849 33.72646277

H 1.29182119 0.89355096 33.81768285

H -1.12610386 3.84589958 34.78045246

H 2.10852554 3.91606515 28.18103470

H 0.60152271 4.76683226 28.00205968

H 4.27424925 4.28714486 29.30103755

H 4.49358246 -0.88345651 28.21007446

H 3.81743809 0.23261383 27.01772799

H 5.47518307 -0.35145755 26.82208527

H -4.75628812 3.44726150 30.65079542

H -3.24995238 2.51421359 30.75985932

H -4.67157722 1.98987878 31.66920065

**(GC/4,7-(NH_2_)_2_phen/CG)mg**

C 5.39542539 3.07499603 31.41234937

N 5.85603073 4.38168573 31.31834342

C 5.26637843 5.29100135 30.33505924

N 4.24573598 4.82164829 29.53402228

C 3.80102365 3.52984672 29.64457631

C 4.38600149 2.62471744 30.60042978

C 7.02034040 4.89018718 32.12136804

O 8.11808938 3.99355943 31.90676475

C 8.35053429 3.15279964 33.07630931

C 7.26798077 3.55123952 34.11498751

C 6.73003076 4.89936871 33.62476166

O 7.90938447 3.78426125 35.38921241

P 7.80676242 2.47769034 36.39207365

O 8.11062377 1.16112751 35.72830062

C 8.29597669 1.69075000 32.59783538

O 7.18796227 0.99382363 33.16324637

O 5.71200810 6.43078994 30.27856623

N 2.78479111 3.15353029 28.81287671

O 6.17445886 2.45010834 36.76380613

C 5.74974686 3.35616752 37.79102570

C 5.94620609 2.77722322 39.20182571

O 4.63650577 2.84928697 39.84690577

C 4.17752413 1.53755631 40.19201366

C 5.46724625 0.72585608 40.41790430

C 6.37247213 1.28135228 39.29927413

N 3.39860287 0.97451514 39.05038117

C 2.57304802 1.67539355 38.16412831

C 2.27402910 0.76474064 37.09401718

N 2.90588164 -0.46791388 37.31411553

C 3.57803982 -0.33609827 38.45098712

N 2.10891932 2.96646144 38.26015503

C 1.26464150 3.33781258 37.24818949

N 0.94509719 2.51107195 36.14825106

C 1.42193053 1.15437228 36.01250748

O 1.07719803 0.55841019 35.00208893

N 0.66696231 4.57464770 37.30106835

O 7.73533882 1.15232142 39.68851584

O 8.59688269 2.76028487 37.67477450

N 3.79345267 2.85223769 34.43585657

C 3.30617366 4.15906005 34.45633795

C 2.58477902 4.71347688 33.33876526

C 2.42856823 6.12032308 33.30276482

N 2.70482305 6.93703980 34.38439942

C 3.26521492 6.36803954 35.46570519

C 3.61402191 5.00455760 35.53110559

C 1.94686500 3.91643966 32.32179957

C 1.16257665 4.49049377 31.36994203

C 1.05055590 5.92297860 31.25597865

C 1.84756720 6.74211151 32.09700950

N 2.04305168 8.09195246 31.87069005

C 1.29615739 8.68294436 30.91549568

C 0.32158963 7.97838621 30.17284284

C 0.19950160 6.59784221 30.31594273

N -0.80805638 5.87637347 29.61625849

C -2.97550300 7.42115858 31.79947414

C -3.89044440 8.47555523 32.45398424

C -3.88481890 8.07853307 33.95868263

O -2.70459803 7.26219175 34.18275916

C -2.05574729 6.94734923 32.92566780

O -3.53456741 9.82854643 32.31352848

P -1.96261748 10.37168711 31.98468425

O -0.98597519 9.54433307 32.77439709

N -1.80804311 5.45764771 32.96606141

C -0.96636452 4.91145025 34.03309839

N -0.74148910 3.54990691 34.06420243

C -1.22672933 2.72680844 33.07869635

C -2.07933067 3.24874079 32.04360398

C -2.34532557 4.59319133 32.02263216

C -5.08702551 7.20290029 34.35288897

O -6.28934332 7.67567911 33.73809982

O -0.46842127 5.67065729 34.86034389

N -0.83328174 1.42215114 33.10532676

O -1.83336326 9.67515749 30.43333647

C -2.39527913 10.40034464 29.34552388

C -1.50877860 11.59217621 28.94689287

O -0.14642981 11.15470124 28.70490414

C 0.15364805 11.10483841 27.30516692

C -0.91065196 11.93044459 26.57822875

C -1.98259827 12.27487765 27.63842054

N 0.17234464 9.67499877 26.89488826

C 1.21359946 8.80462879 27.25447236

C 0.70838571 7.47291932 27.09499093

N -0.62784567 7.52125755 26.65826801

C -0.94059809 8.81295105 26.56643483

N 2.46122336 9.14920204 27.71031081

C 3.23725812 8.08083824 28.07372565

N 2.80954378 6.73718381 27.97448900

C 1.51250997 6.35138617 27.48254501

O 1.24537778 5.15363924 27.52516085

N 4.51978145 8.31385988 28.51905901

O -2.10743844 13.68156963 27.78981312

O -2.03766208 11.84306496 31.90602795

Na 9.64094386 0.85845216 38.46196465

Na 0.17478275 7.90670890 33.67389870

H 3.47162194 7.02575913 36.32362650

H 2.06931450 2.83395117 32.36346286

H -4.98702716 6.17208675 33.96179483

H -5.21518084 7.15937197 35.44827848

H -3.76352683 8.96152777 34.62431372

H -4.92655965 8.45128483 32.02774061

H -3.60308632 6.61614616 31.36348194

H -2.42538101 7.82775511 30.91099728

H -1.02261156 7.46967510 32.99519753

H -1.21987883 0.74488471 32.46982934

H -0.19488385 1.06700898 33.84479793

H -2.49710444 2.59050568 31.28877833

H -2.98669262 5.03152642 31.23451318

H -3.42162570 10.74843395 29.58585700

H -2.44427427 9.63726856 28.54413983

H -1.39717532 12.31026072 29.80537792

H -3.00674427 11.98240466 27.32063227

H -1.34522481 11.41765925 25.70387816

H -0.47585969 12.86316123 26.16533371

H 1.20240364 11.49416528 27.23476399

H -1.88760900 9.21892519 26.24565071

H 3.42244200 5.97061812 28.39286972

H 4.78339295 9.27167845 28.72633228

H 5.01101123 7.58642659 29.07329868

H 8.12306098 1.64669676 31.50261894

H 9.23164070 1.15801720 32.84393087

H 9.36855352 3.44116203 33.42081991

H 6.44762907 2.79181788 34.18993583

H 5.66141012 5.04994536 33.86535252

H 7.26112924 5.73272723 34.13227961

H 7.34696302 5.86964193 31.68453067

H 2.41685542 2.21932737 28.82723461

H 2.31515679 3.84464282 28.19445054

H 4.04339481 1.59757687 30.67008591

H 5.86373829 2.40838423 32.16366443

H 4.65661698 3.48359628 37.58118966

H 6.23840321 4.34571145 37.68923656

H 6.58264239 3.44142107 39.83083698

H 6.20013283 0.72830772 38.33907673

H 5.34072209 -0.36716110 40.37753597

H 5.89387835 0.94042928 41.41925052

H 3.50514000 1.69514627 41.06527907

H 4.17106114 -1.09617128 38.93598656

H 0.29325041 2.88256841 35.39026710

H 0.95739762 5.22892858 38.01640003

H 0.19532210 4.95929793 36.46837260

H 4.09948793 4.61434335 36.42525319

H 4.22524120 2.48756986 35.28580375

H 0.62133911 3.87525859 30.64990371

H -1.24216052 6.41574942 28.86001329

H -0.32706417 8.55241446 29.49062561

H 1.48930916 9.75408907 30.70267030

H -1.34806510 14.06271954 28.28483638

H 7.40427123 0.78287726 34.14242631

H 8.33533044 1.71293734 38.98439120

H -6.51055889 8.57579972 34.06115415

H 3.29626835 2.15366924 33.89820054

H -0.47867881 4.99145162 29.22282024

**(GC/4,7-(OH)_2_phen/CG)mg**

C 1.37810864 6.24882971 27.43703240

C 0.56238628 7.33581700 26.96972724

C 1.08167919 8.66734634 26.99642919

N 2.35817037 9.04996681 27.32671034

C 3.15693764 8.01377601 27.72830245

N 2.71437492 6.67853238 27.80389756

N -0.79839078 7.36481864 26.61824342

C -1.12039612 8.64776432 26.47071531

N 0.01832038 9.52522610 26.65799339

C 0.05386942 10.95281163 27.04412713

O -0.05413981 11.00751386 28.48283515

C -1.34604482 11.51278633 28.89955149

C -1.97824332 12.19515701 27.66046620

C -1.09081840 11.78595507 26.46442732

C -2.18230372 10.33854579 29.42582046

O -1.40547939 9.58961810 30.36064030

P -1.39563359 10.16398032 31.97957514

O -1.34365044 11.63328464 32.03014322

O -2.02532104 13.60691875 27.80685347

N 4.47698419 8.27962227 28.03674689

O 1.13247781 5.06579251 27.61622485

O -3.00849731 9.73227634 32.25987068

C -3.48161900 8.40481223 32.27629200

C -2.54224124 7.27686979 31.79601679

C -1.85203015 6.78691378 33.07457560

O -2.55115032 7.35223258 34.20465672

C -3.75371361 8.03219855 33.76252004

C -4.95015809 7.09364268 33.98074016

O -5.23645757 6.29339036 32.82014569

N -1.89387171 5.29627609 33.29722527

C -1.03567242 4.73114726 34.33723392

N -1.11458949 3.38028156 34.59167150

C -1.97130766 2.56788101 33.88952757

C -2.91754209 3.14005817 32.96643866

C -2.86947533 4.48619975 32.72130236

O -0.25521925 5.46026707 34.94876391

N -1.88862228 1.23001008 34.13679837

O -0.45490392 9.19269083 32.63760057

O 0.19919053 7.90014092 29.72073229

C 1.21542345 7.81094737 30.59185308

C 1.38673916 6.52202134 31.20641868

C 2.38091095 6.42665340 32.22253823

N 3.22612906 7.47332527 32.53368461

C 3.09191693 8.63248133 31.84984962

C 2.09215806 8.85760657 30.88448934

C 2.44592263 5.17863892 33.00399285

C 1.63072924 4.08775850 32.60721611

C 0.70637073 4.21024534 31.50984897

C 0.58801568 5.38242760 30.83309125

N 3.28817142 5.11204361 34.09580211

C 3.42483792 3.93786106 34.73068743

C 2.73095712 2.76568142 34.35811319

C 1.80214026 2.86220620 33.33028246

O 1.02487452 1.81644017 32.93588960

O 0.53638883 0.32220974 35.41418062

C 1.05002854 0.91279367 36.36420587

C 2.02483165 0.50074600 37.32537660

C 2.53142209 1.43109203 38.30039069

N 2.14882509 2.74953211 38.41307044

C 1.19408871 3.13689318 37.51971640

N 0.63737482 2.27912786 36.54597084

N 3.47921270 0.73156730 39.04915584

C 3.51048021 -0.60191954 38.48277789

N 2.65300975 -0.74404373 37.47728182

N 0.70118130 4.42681672 37.57294150

C 4.36402324 1.26130194 40.13212955

O 4.65156509 2.63598864 39.88054311

C 5.91236919 2.78172066 39.15620031

C 6.52934654 1.35454266 39.08896392

C 5.72772041 0.54841251 40.13039827

C 5.55975709 3.43627903 37.80863057

O 6.25166351 2.84353713 36.70443898

P 7.87341540 3.18712687 36.50058888

O 7.83440747 4.54740520 35.56895764

C 7.24709254 4.30582308 34.26855145

C 8.37143337 4.03009811 33.22971979

O 8.09994653 4.90912872 32.09797747

C 6.87926834 5.63765242 32.29475377

C 6.60573951 5.61595344 33.80026453

O 7.89748274 1.37331920 39.48963966

C 8.41401291 2.59702267 32.66550665

O 7.43967541 1.75835659 33.28018866

N 5.80154028 4.95841022 31.49557790

C 5.23006215 5.68560293 30.36556089

N 4.21975203 5.08963116 29.63461199

C 3.76937167 3.83357579 29.94795020

C 4.36428052 3.08894275 31.02860580

C 5.36135081 3.66801740 31.76730691

O 5.66424172 6.80775655 30.12534858

N 2.76765151 3.31271949 29.17683235

O 8.51327310 3.47064698 37.86375909

O 8.44824940 1.97798746 35.79878282

Na 9.86827525 1.57740554 38.38464008

Na 0.55277581 7.44989403 33.57795373

H 4.12993563 3.91320464 35.57653475

H 0.12853867 3.32263896 31.23878326

H -4.69882313 6.33333580 34.75177528

H -5.85681854 7.64291974 34.28491014

H -3.80576838 8.93113393 34.41951488

H -4.41168626 8.45682224 31.66563459

H -3.14943505 6.49475828 31.28932385

H -1.83150831 7.60897955 30.99269659

H -0.75881219 7.13110088 33.20257928

H -2.48679163 0.57056592 33.66758477

H -1.11342509 0.84365734 34.70831053

H -3.66331696 2.51463928 32.48445361

H -3.60274184 4.97947012 32.04984300

H -3.13065367 10.69061339 29.88019328

H -2.40412715 9.59455284 28.63422047

H -1.09487571 12.23262187 29.72238528

H -3.05029015 11.94276092 27.50715039

H -1.66693293 11.25238317 25.68894718

H -0.70446618 12.68888304 25.94996169

H 1.08432291 11.34527351 26.83871782

H -2.08416987 9.03885518 26.18969974

H 3.34564892 5.96633473 28.28107064

H 4.75571811 9.25292805 28.09641031

H 4.98563167 7.63830933 28.67573905

H 8.13932408 2.59678002 31.58982671

H 9.41618517 2.15061328 32.78774228

H 9.36264241 4.36536726 33.60397555

H 6.49177233 3.47667871 34.30425765

H 5.52876322 5.71051118 34.05366491

H 7.08104880 6.48200570 34.30673725

H 7.06564316 6.64616532 31.84438581

H 2.31035381 2.45748711 29.45079013

H 2.25488294 3.90901717 28.49701587

H 4.03430107 2.07716991 31.24720296

H 5.83798968 3.11429479 32.60134235

H 4.49093343 3.23916905 37.55812157

H 5.71726222 4.53191526 37.83729542

H 6.49982956 3.47185394 39.80704555

H 6.44334244 0.89635697 38.07013151

H 5.68528479 -0.52732765 39.89548325

H 6.20344161 0.61676128 41.13134308

H 3.79770446 1.26593977 41.09302591

H 4.16155693 -1.36482827 38.88148863

H -0.06946536 2.67539112 35.85389893

H 1.16699937 5.09013267 38.18231072

H 0.23953013 4.81773406 36.74065881

H 2.94660665 1.82324962 34.85738379

H 1.00443557 1.07799506 33.63997506

H -0.07284917 5.48488539 29.95764963

H -0.08155924 8.89497341 29.51334967

H 1.99926187 9.82549904 30.38061190

H 3.83614832 9.41530536 32.07946783

H -1.15959133 13.97265710 28.09447432

H 7.74829721 1.54858655 34.23320271

H 8.40359768 2.15554466 38.93581502

H -5.79959911 6.78995245 32.19273907

**(GC/4,7-(NH_2_)_2_phen/CG)MG**

C -3.20873230 5.43536145 32.71546174

N -2.80904833 5.96429976 33.94000783

C -1.80465924 5.26801925 34.73401960

N -1.33156747 4.05725489 34.28238627

C -1.76855347 3.51823113 33.09596359

C -2.71148918 4.24000705 32.27492000

C -3.33456658 7.26756001 34.46906041

C -2.78136917 8.45668821 33.68174698

C -3.82956849 8.68390429 32.58707291

C -5.15516551 8.17992784 33.21922826

O -4.76018798 7.27079155 34.29437039

O -3.92948956 10.10576459 32.37456292

P -4.17064195 10.50153465 30.78387374

O -5.44798115 9.86780161 30.25499713

C -6.08588821 7.38207102 32.28991145

O -5.53172109 7.21745154 30.98952913

O -1.42297698 5.81064087 35.77007178

N -1.33704306 2.26536706 32.76593402

O -2.84150005 9.69205401 30.18187172

C -2.24873171 10.16709360 28.97195066

C -1.41717646 11.43170256 29.23623352

O -0.10456419 11.02947622 29.70243885

C 0.87366761 11.15596348 28.64976670

C 0.33261003 12.22449663 27.69312844

C -1.18454504 12.30417690 27.97217179

N 1.02587645 9.83991282 28.00102449

C 1.69512486 8.75650716 28.59606765

C 1.29474440 7.59134888 27.87086428

N 0.38651312 7.93854295 26.86129485

C 0.21305632 9.25392596 26.95169495

N 2.58754534 8.81259585 29.63804242

C 3.14369651 7.59123988 29.94596226

N 2.78879150 6.39467332 29.30623525

C 1.80661511 6.29789326 28.23941028

O 1.56446943 5.17833570 27.83573774

N 4.05077498 7.52780303 30.97998497

O -1.60668187 13.64427122 28.17791155

O -4.11186346 12.00504632 30.60163623

N 2.56949922 2.70583881 29.09647021

C 3.72107634 2.79036658 29.82242318

N 4.18755623 4.04721409 30.13040057

C 5.41258018 4.23159551 30.72107831

N 6.25338043 3.06070542 30.95356242

C 5.74552075 1.78476736 30.71571468

C 4.49645943 1.62348312 30.18250794

C 7.63437708 3.30628260 31.47826408

O 8.51982009 2.35092113 30.87312820

C 8.99347762 1.37226228 31.85259786

C 8.17045677 1.64594039 33.13633008

C 7.70485876 3.10192784 32.99688050

O 8.99714319 1.52378130 34.29297631

P 8.64946422 0.11365543 35.19493605

O 9.39104667 0.48010116 36.47966548

C 8.79329576 -0.02357107 31.23303651

O 7.75045418 -0.74728818 31.87346220

O 5.85263211 5.32448374 31.07421695

O 7.01172089 0.14855478 35.40184525

C 6.35951977 1.32717617 35.88855107

C 6.60204704 1.54994449 37.38592713

O 5.44108914 2.27427848 37.87305330

C 4.67428438 1.44393607 38.77902568

C 5.65067473 0.39762929 39.32498918

C 6.70809783 0.23854959 38.21779580

N 3.57387159 0.84303207 37.99610077

C 2.55221058 1.60605795 37.40425358

C 1.95056827 0.77352597 36.41307064

N 2.59172460 -0.47496117 36.37644371

C 3.56137694 -0.42182671 37.28313256

N 2.20918764 2.90485036 37.70001907

C 1.20798946 3.40211069 36.90341066

N 0.56101306 2.65661291 35.90614989

C 0.90127022 1.28393958 35.57332246

O 0.29015114 0.78531682 34.64810597

N 0.88807090 4.74102008 37.02535656

O 7.98534898 0.14728686 38.83904635

O 8.94544225 -1.06957871 34.34448986

C 3.48052867 2.26107377 33.99795291

N 1.88041241 2.60029277 32.20505798

C 1.81108863 3.93976051 32.53623312

C 2.54958047 4.48718051 33.61512285

C 3.40315671 3.60388713 34.37077512

C 2.41265950 5.88561197 33.92782908

C 1.60674938 6.69896531 33.19623101

C 0.84941092 6.19535399 32.08057196

C 0.93036237 4.81968096 31.74761347

N 0.20147310 4.27187426 30.70999146

C -0.62813312 5.07372749 30.02047740

C -0.77784875 6.45177813 30.27634257

C -0.01528509 7.03704169 31.28950217

C 2.69592075 1.80658341 32.91739261

N -0.14841201 8.40626661 31.58002586

N 4.20641410 4.08449227 35.42519688

Na 11.34072097 1.56194857 36.06501301

Na -6.35381640 12.56073761 29.99999711

H 2.97240051 6.28301013 34.77434579

H -6.21292340 6.34544497 32.66756477

H -7.07586541 7.86453198 32.21189489

H -5.69138543 9.00568804 33.73482178

H -3.56559451 8.12553969 31.65214320

H -1.76729431 8.27905249 33.26936768

H -2.67912559 9.36086460 34.31397345

H -3.16724061 7.29064416 35.57455748

H -1.31315661 1.98836320 31.79206894

H -0.64962548 1.77760662 33.36875458

H -3.02384204 3.82779140 31.31916499

H -3.93991594 6.00973074 32.11789583

H -2.99999848 10.30090114 28.17319844

H -1.59497345 9.30669745 28.69520257

H -1.81984143 12.03081829 30.09183371

H -1.79809382 11.99937941 27.09670401

H 0.55189653 12.01067804 26.63212109

H 0.81227699 13.20411134 27.88483317

H 1.81820420 11.40967573 29.19341665

H -0.40183424 9.86461036 26.31287491

H 3.24805422 5.48216252 29.62584212

H 4.41723609 8.39024055 31.35717913

H 4.64274010 6.68132152 31.09806805

H 8.45719181 0.06896250 30.17877737

H 9.72986983 -0.60821399 31.27071923

H 10.06921146 1.61040052 31.97693921

H 7.27844953 0.95831091 33.17886762

H 6.73611419 3.29213978 33.50068612

H 8.41573201 3.80756318 33.46398144

H 7.97433009 4.30741049 31.11306916

H 2.00563782 1.86852422 29.14395714

H 2.04167129 3.56588251 28.86514979

H 4.09009484 0.63019335 30.01046938

H 6.38869825 0.92242336 30.96962459

H 5.28413193 1.08980311 35.68059141

H 6.63177676 2.22058449 35.29420956

H 7.44891835 2.23705865 37.59825970

H 6.54505426 -0.67148724 37.59733687

H 5.17425738 -0.55811555 39.59951900

H 6.14249994 0.75927881 40.25359318

H 4.25290285 2.17132501 39.51455210

H 4.25651265 -1.21046306 37.52633162

H -0.17941049 3.13680411 35.29840575

H 1.17359711 5.22375420 37.86736403

H 0.03070392 5.10743520 36.56122924

H -1.25622619 14.00994383 29.02013745

H 8.09215220 -1.11231252 32.77249881

H 8.69162976 0.07570608 38.10886024

H -5.56588818 8.11685019 30.49648091

H -1.18892247 4.60205437 29.20199821

H -1.46232748 7.04090501 29.67078926

H 0.69534845 8.88918144 31.87034631

H 1.51068594 7.75361040 33.45308279

H 2.71638709 0.74504142 32.63173845

H 4.10191821 1.54980108 34.54358873

H 3.88696716 4.94394343 35.86204149

H 4.47117067 3.40291479 36.14262421

H -0.69049485 8.97177003 30.92024827

**(GC/4,7-(OH)_2_phen/CG)MG**

C -1.19981547 4.45533766 29.28133395

C -1.53206867 5.81400618 29.47392773

C -0.78578169 6.53293495 30.39779737

C 0.27226188 5.92005709 31.13311154

C 0.49098011 4.53635556 30.89968942

N -0.23373863 3.82284567 29.97179765

C 1.53608177 3.84698911 31.67406030

C 2.39341731 4.62576883 32.49208714

C 2.13520069 6.02834604 32.68980477

C 1.10151652 6.64415884 32.06113985

N 1.63927110 2.47626057 31.59685950

C 3.58142852 2.55416976 33.05342490

C 3.47218580 3.93465386 33.12590416

C 2.60496426 1.85858999 32.30322662

O -1.17100009 7.82824613 30.59306954

O 4.45102258 4.58148871 33.82839363

O 0.91630158 1.20342885 34.35515288

C 1.47143619 1.74854122 35.29406686

C 2.66207775 1.41121629 36.01921932

C 3.08615797 2.20956163 37.12374472

N 2.46715797 3.33972882 37.59986269

C 1.35633884 3.70262296 36.87632724

N 0.86940467 2.97535456 35.77347515

N 4.26046747 1.60836430 37.61276157

C 4.53003829 0.50192962 36.70800633

N 3.58044945 0.37052055 35.79186505

N 0.65413731 4.81956425 37.26246382

C 5.11246988 2.21088195 38.64607235

O 5.96580961 3.21396754 38.05153504

C 7.27795338 2.66597069 37.77801465

C 7.48131785 1.58209246 38.87547442

C 6.05803999 1.19551344 39.32427716

C 7.28349804 2.14095180 36.32935333

O 7.41722195 0.71897824 36.35730924

P 8.60398345 0.00275117 35.34613854

O 8.01110620 -1.19956340 34.70487113

O 8.21792154 2.14173668 39.98174291

O 8.82256866 1.23459168 34.23530464

C 7.87599950 1.19298272 33.15174673

C 8.55913963 0.51830243 31.93575354

O 8.29453963 1.38225074 30.78528714

C 7.62413460 2.58154157 31.18207625

C 7.61291747 2.63824010 32.71471181

C 8.03265143 -0.88714389 31.60208384

O 6.79154460 -1.15037572 32.25084822

N 6.23327282 2.52579491 30.60932389

C 5.58350809 3.79367707 30.31703795

N 4.36492746 3.77958794 29.67876348

C 3.75096448 2.59228543 29.34783120

C 4.34108718 1.33739661 29.74591740

C 5.56701444 1.33109990 30.35698639

O 6.15927048 4.82216494 30.67490504

N 2.61149125 2.65362845 28.59847562

O 9.77968698 -0.00662201 36.29227369

O 1.76464033 5.36427140 27.64103003

C 2.03118400 6.38772473 28.23377299

C 1.43769005 7.69882455 28.21805511

C 1.86355672 8.69016985 29.14845062

N 2.88713883 8.58604771 30.05366128

C 3.52657611 7.36541379 30.01580141

N 3.13617675 6.31199743 29.17990333

N 1.04736847 9.82523026 28.92705915

C 0.14779996 9.42675691 27.85115936

N 0.39361000 8.19824680 27.42110747

N 4.56867790 7.14919194 30.89630826

C 0.79684824 10.80807813 29.98578526

O -0.27821955 10.28922446 30.82157544

C -1.52368234 10.99199949 30.56796252

C -1.10856992 12.37361503 29.99769098

C 0.32918472 12.18153453 29.48088317

C -2.41105784 10.23162607 29.56077977

O -3.64315626 10.94321822 29.55109620

P -4.95826746 10.22352495 30.38518540

O -5.76685447 11.45754851 30.64718750

O -1.18852066 13.37321954 31.02432639

O -5.29785131 8.90706039 29.79062580

O -4.14037925 9.98797602 31.86820452

C -3.99021012 8.62413396 32.25180486

C -2.75523477 8.49977871 33.15760117

C -3.15436051 7.43241264 34.18457097

O -4.58390596 7.43455152 34.26180984

C -5.17484589 8.19171022 33.15915960

N -2.69777149 6.06509625 33.76810192

C -1.67932802 5.41256838 34.58416240

N -1.23726790 4.16118781 34.22135295

C -1.73084825 3.53581697 33.09847671

C -2.74721846 4.17297541 32.29711774

C -3.21715289 5.40642771 32.65652728

C -6.22552163 7.27652861 32.50993114

O -5.76243227 6.68168150 31.30232739

O -1.24347931 6.03947788 35.55004118

N -1.26921964 2.28331249 32.81890214

Na 9.59150192 0.19515753 38.45072379

Na -3.96627073 13.09195023 31.18015195

H 2.78633169 6.57478786 33.37295134

H -6.43231026 6.40858158 33.17258769

H -7.16408061 7.82626182 32.31644100

H -5.65112035 9.06657027 33.65456799

H -3.89253531 7.95736933 31.35228071

H -1.85007488 8.22765463 32.58097062

H -2.52933990 9.45888052 33.65670665

H -2.80261797 7.61199199 35.23270423

H -1.36067433 1.90786992 31.88135215

H -0.49035085 1.87527563 33.37271089

H -3.15019428 3.66250991 31.42573572

H -4.01867802 5.91438060 32.07793071

H -2.03561217 10.27977962 28.51430157

H -2.50829365 9.14818292 29.81326969

H -1.99371599 11.02391307 31.58650332

H -1.80205345 12.76591482 29.20927156

H 0.37645690 12.25461213 28.37502483

H 1.00233214 12.98691187 29.83326478

H 1.67766455 10.84015132 30.68008086

H -0.59734060 10.11106124 27.47080900

H 3.62252603 5.35928912 29.27741659

H 4.99730619 7.95400054 31.33366431

H 5.17916214 6.31362523 30.76843250

H 7.80616119 -0.97489968 30.52069475

H 8.77158119 -1.65840435 31.89065986

H 9.66804338 0.53148877 32.03352490

H 6.92346086 0.67623868 33.43835586

H 6.66610413 3.04411786 33.12140655

H 8.41111036 3.30837890 33.09368522

H 8.16582071 3.42102826 30.68038250

H 1.95923665 1.87853000 28.60763539

H 2.18773023 3.56390100 28.36803758

H 3.81491190 0.40526356 29.55389701

H 6.06363450 0.39498346 30.67381991

H 6.28865882 2.34753316 35.86061239

H 8.05697293 2.64894073 35.71318315

H 7.93739068 3.55813359 37.89981570

H 8.17334919 0.72077184 38.55957520

H 5.78522305 0.15229425 39.06396369

H 5.94753335 1.24805688 40.42719590

H 4.47094468 2.80939983 39.34462244

H 5.42146445 -0.10730423 36.80423476

H 0.02224200 3.34555910 35.23665269

H 1.02856637 5.41042992 37.98990932

H -0.08093647 5.22355272 36.64888888

H -0.44507989 13.29090166 31.66445520

H 6.97917717 -1.33995360 33.24151363

H 7.76478858 2.93103778 40.35999578

H -5.77371501 7.37698201 30.55548710

H -1.73483287 3.85537222 28.53271577

H -2.32747269 6.29140585 28.90280415

H -0.42832127 8.45740669 30.86629047

H 0.88491556 7.69947880 32.22859364

H 2.60904780 0.75741850 32.27489560

H 4.36505540 2.01300587 33.58067229

H 4.37487294 5.57114592 33.74438350
